# Construction of a Chinese traditional instrumental music dataset: A validated set of naturalistic affective music excerpts

**DOI:** 10.3758/s13428-024-02411-6

**Published:** 2024-05-03

**Authors:** Di Wu, Xi Jia, Wenxin Rao, Wenjie Dou, Yangping Li, Baoming Li

**Affiliations:** 1 Institute of Brain Science and Department of Physiology, School of Basic Medical Sciences, Hangzhou Normal University, Hangzhou, 311121 China; 2 Zhejiang Philosophy and Social Science Laboratory for Research in Early Development and Childcare, Hangzhou Normal University, Hangzhou, 311121 China; 3 School of Foreign Studies, Xi’an Jiaotong University, Xi’an, 710049 China

**Keywords:** Chinese traditional instrumental music, Dataset, Dimensional emotion, Discrete emotion

## Abstract

Music is omnipresent among human cultures and moves us both physically and emotionally. The perception of emotions in music is influenced by both psychophysical and cultural factors. Chinese traditional instrumental music differs significantly from Western music in cultural origin and music elements. However, previous studies on music emotion perception are based almost exclusively on Western music. Therefore, the construction of a dataset of Chinese traditional instrumental music is important for exploring the perception of music emotions in the context of Chinese culture. The present dataset included 273 10-second naturalistic music excerpts. We provided rating data for each excerpt on ten variables: familiarity, dimensional emotions (valence and arousal), and discrete emotions (anger, gentleness, happiness, peacefulness, sadness, solemnness, and transcendence). The excerpts were rated by a total of 168 participants on a seven-point Likert scale for the ten variables. Three labels for the excerpts were obtained: familiarity, discrete emotion, and cluster. Our dataset demonstrates good reliability, and we believe it could contribute to cross-cultural studies on emotional responses to music.

**Supplementary Information:**

The online version contains supplementary material available at 10.3758/s13428-024-02411-6.

## Introduction

Music has been omnipresent among human cultures since ancient times (Vuust et al., 2022). Enjoying and playing music can shape brain structure and functions (Benz et al., 2016; Burunat et al., 2016; Strait et al., 2013; Strait & Kraus, 2014; Yurgil et al., 2020). A particularly important feature of music is that it can evoke strong emotions (Juslin & Västfjäll, 2008), from happiness to sadness, all of which are associated with some sort of hedonism and pleasure (Mas-Herrero et al., 2018; Sachs et al., 2015; Zentner et al., 2008). People consciously or unconsciously use music to change, create, maintain, or enhance their mood in daily life (van Goethem & Sloboda, 2011). In recent decades, a great many studies have been dedicated to uncovering the processing of music emotion perception (Chan & Han, 2022; Vuilleumier & Trost, 2015; Zatorre et al., 2007).

It is well documented that emotional responses to music are the products of both musical features (melody, harmony, rhythm, etc.) and cultural cues (Balkwill & Thompson, 1999; Brattico & Pearce, 2013; Juslin & Västfjäll, 2008; Juslin, 2013a). The cue-redundancy model (Balkwill & Thompson, 1999) illustrates the perception of emotion in music by an interplay of music-inherent cues and culture-specific cues. Music-inherent cues can be explained as psychophysical cues to all tonal systems. For instance, sad music typically features fairly constant pitch and dynamics, minor harmonies, and slow tempos, whereas happy music often has relatively constant pitch and dynamics, major keys, and upbeat rhythms (Krumhansl, 2002). On the other hand, culture-specific cues are clarified as both the living environments and subjective interpretation of the environments (Geertz, 1973). The BRECVEMA (Brain stem reflex, Rhythmic entertainment, Evaluative conditioning, Contagion, Visual imagery, Episodic memory and Aesthetic judgment) framework outlines nine mechanisms for interpreting the emotional responses to music and highlights the significant influence of culture on music emotion perception (Juslin & Västfjäll, 2008; Juslin, 2013a).

The perception of music emotion involves both universal elements common across cultures and culturally specific elements varying from one culture to another. Fritz et al. (2009) reported that both Mafa and Western participants could recognize the basic emotions of Western music, while the impact of consonance on perceived pleasantness seemed to be culturally influenced in Mafa and Western music. Argstatter (2016) revealed that participants from Europe and Asia could universally recognize happy and sad Western music, but consistently displayed confusion among emotions of anger, disgust, fear, and surprise. Other studies, such as those by Hu and Lee (2012; 2016), reported the impact of cultural background on mood judgments in both Western and Chinese music. Furthermore, the Stereotype Theory of Emotion in Music (STEM) emphasized that music emotions are perceived through cultural stereotypes (Susino & Schubert, 2017; 2018). These studies have highlighted the crucial role of cultural factors in the perception of emotion in music.

Our current knowledge about the emotion processing of music is based mostly on studies on Western music (Hamada et al., 2018; Vuust et al., 2022). As an integral part of world music, Chinese traditional instrumental music, with its long history dating back 7000–8000 years, has considerable research value. From the cultural origin aspect, Chinese traditional instrumental music is deeply influenced by Confucian and Taoist philosophies, emphasizing unity between humans and nature (Hao, 2023). On the other hand, Western music reflects the emotional spectrum of the individual, articulating personal experiences (Zabulionite et al., 2019). From the musical element aspect, Chinese traditional instrumental music relies on the pentatonic scale, while Western music utilizes the 12-tone average (Zhang et al., 2022). Chinese musical instruments are predominantly made of natural materials such as wood and bamboo, whereas Western musical instruments often employ metal and hardwoods (Hao, 2023). The timbre of Chinese instruments is generally rougher than that of Western instruments, and the timbre similarity is lower (Jiang et al., 2020). Chinese traditional instrumental music emphasizes a single melody and linear beauty, while Western music focuses on multidimensionality (harmony and polyphony) (Zabulionite et al., 2019). Thus, it is important to explore how we perceive the emotions of Chinese traditional instrumental music.

In the field of music emotion studies, the dimensional model and discrete model have been widely used (Eerola & Vuoskoski, 2011). In the dimensional model, the two-dimensional circumplex model is frequently adopted. This model evaluates emotions on varying degrees of valence and arousal (Russell, 1980). However, it has been criticized for its inability to effectively distinguish between similar emotions within the same valence–arousal quadrant, such as fear and anger, and for possibly missing additional emotion dimensions (Fuentes-Sánchez et al., 2021; Schubert, 2013). In the discrete model, the basic emotion model has frequently been utilized (Ekman, 1992), and some emotional terms are frequently substituted with more appropriate music emotion concepts (Vieillard et al., 2008). However, its effectiveness in capturing the aesthetic emotions of music has been debated. This debate has led to the development of music-specific discrete models like the Geneva Emotional Music Scale (GEMS) (Zentner et al., 2008).

Many studies have been carried out to uncover the different uses between the dimensional and discrete models. Some researchers (Eerola & Vuoskoski, 2011; Korsmit et al., 2023) have reported that the discrete model is less dependable, with ambiguous emotion excerpts, than the dimensional model, and the consistency of ratings is highest when the dimensional model is employed (Vuoskoski & Eerola, 2011). Berrios et al. (2015) proposed that the two models produce similar accounts for the magnitude of experience of mixed emotions. Cowen et al. (2020) found that specific feelings such as “triumphant” were better preserved across different cultures than valence and arousal. Such controversial results have triggered a trend of a combined use of the dimensional and discrete models (Eerola & Vuoskoski, 2013), since the core emotion often demonstrates dimensionality, but subjective understanding or evaluation of emotion might be articulated using categorical descriptions (Russell, 2003; Barrett, 2006).

Familiarity is an important factor in the emotional feelings of the listener. Familiarity influences both perception and recognition of music emotion. Familiarity regulates hedonic brain responses. Positive emotions can be evoked by increasing the likeability of music through repetition and familiarity (Freitas et al., 2018). It is known that familiar music and unfamiliar music are perceived differently. Predictive brain mechanisms for familiar music depend on both schematic (implicit knowledge about the encultured rules of music) and veridical expectations (factual knowledge about specific pieces of music) (Hansen et al., 2017). On the other hand, emotion recognition is more accurate for culturally familiar versus unfamiliar music in both basic and non-basic emotions (Laukka et al., 2013). Listeners who are more familiar with the genre report fewer stereotypical emotions than those who are less familiar (Susino & Schubert, 2018).

Various affective music datasets for exploring emotion perception in music have been reported (for an overview see Table 1). As shown, both the dimensional (Belfi & Kacirek, 2021; Koh et al., 2022; Li et al., 2012; Lepping et al., 2016; Imbir & Gołąb, 2017; Rainsford et al., 2018) and discrete (Hill and Palmer, 2010; Xu et al., 2017; Vieillard et al., 2008) emotion models have been employed to categorize music emotion, yet only a few studies have employed both models for music classification (Eerola & Vuoskoski, 2011; Xie & Gao, 2022). Most of the datasets use Western music and pop music (Belfi & Kacirek, 2021; Eerola & Vuoskoski, 2011; Imbir & Gołąb, 2017; Koh et al., 2022; Li et al., 2012; Lepping et al., 2016), and only three studies utilize Chinese traditional instrumental music (Li et al., 2012; Xie & Gao, 2022; Xu et al., 2017). Li et al. (2012) and Xu et al. (2017) constructed emotional music datasets containing Chinese traditional instrumental music. However, these datasets include mixed music pieces from different musical genres but small numbers of Chinese traditional instrumental music pieces. Moreover, these datasets are published in Chinese, limiting their accessibility and utilization. Xie and Gao (2022) constructed a dataset to classify the aesthetics of Chinese traditional instrumental music. They primarily employed machine learning for classification and did not systematically investigate the broader emotional properties. Moreover, they used only 20 participants to rate music excerpts, potentially impacting data credibility.

**Table 1:** Sample of data collections of emotional music dataset

References	Name /Description of the data collection	Stimuli						Validation procedure		Category
		Genres	Duration	Playing mode	Rating type (in terms used by authors)	*N*	Familiarity	Sample/Country	Measures	
Koh et al., 2022	Music Emotion Recognition with Profile information (MERP)	Pop music	Full-length	Naturalistic music	**Emotion** Dimensional model: Valence; Arousal **Others:** Demographic information; Listening preferences; Musical experience	50	Not mentioned	447 raters from Singapore	Label valence–arousal (VA) values in a two-dimensional VA graph while listening to the stimuli in real time	Low valence and low arousal; Low valence and high arousal; Mid valence and mid arousal; High valence and low arousal; High valence and high arousal
Xie & Gao, 2022	A dataset for aesthetic classification of Chinese traditional music	Chinese traditional music (folk, rap, song–dance, opera and instrumental music)	20–30 s	Naturalistic music	**Emotion** Dimensional model: Valence; Arousal Discrete model (aesthetic category): LingDong, JiYang, QingRou, ShenChen, BeiShang	500	Not mentioned	20 raters from China	9-point scale	LingDong, JiYang, QingRou, ShenChen, BeiShang
Belfi & Kacirek, 2021	Famous Melodies Set	Children’s; Patriotic; Movie/TV; Christmas; Religious; Classical; Pop; or “other” music	13.31 s (Range: 6–37 s;)	Single-line melody in a piano keyboard timbre	**Emotion** Dimensional model: Valence; Arousal **Others:** Age of acquisition; Familiarity	107	Familiar	397 raters from the USA	5-point scale	Highly familiar; Less familiar
Rainsford et al., 2018	MUSOS (MUsic Software System) Toolkit	Self-composed using a seven-note scale	Half eight-note length, half 16-note length	Single-line melody in a piano keyboard timbre	**Emotion** Dimensional model: Valence; Distinctiveness	156	Novel, and highly distinctive	36 raters from Australia	6-point scale	Not mentioned
Imbir & Gołąb, 2017	Affective reactions to music	Pop; Rock; Jazz; Rap/R&B; Electronic music; and Classical music	15 s	Naturalistic music	**Emotion** Dimensional model: Valence; Arousal; Dominance; Origin; Subjective significance; Imageability	120	Not mentioned	50 raters from Poland	9-point SAM scales	Not mentioned
Lepping et al., 2016	Emotionally Provocative Music	Western instrumental classical music	10 s	Solo to full orchestra	**Emotion** Dimensional model: Valence; Arousal	36	Not mentioned	24 raters form the USA	Rated on the valence–arousal circumplex model	Positive; Negative
Xu et al., 2017 (Chinese Publication)	TCM Emotion Music Treatment Database	Chinese traditional music; Classical; Pop music performed by Chinese traditional instruments	Full-length	Naturalistic music	**Emotion** Discrete model: Calm; Refreshing; Pleasure; Detached; Releasing; Encouraging; Expressing; Pouring **Others:** Degree of favorite; Familiarity	120	Moderate familiarity	37 raters from China	Not mentioned	Calm; Refreshing; Pleasure; Detached; Releasing; Encouraging; Expressing; Pouring
Li et al., 2012 (Chinese Publication)	Standard music stimuli for emotional research in China	Classical; Pop; Chinese traditional music	60 s (Range: 50–70 s)	Naturalistic music	**Emotion** Dimensional model: Valence; Arousal; Expression.	78	Not mentioned	160 raters from China	9-point scale	Positive; Neutral; Negative
Eerola & Vuoskoski, 2011	Set of stimuli consisting of unfamiliar, thoroughly tested and validated non-synthetic music excerpts	Film music	(Range: 10–30 s)	Naturalistic music	**Emotion** Dimensional model: Valence; Tension arousal; Energy arousal Discrete model: Happy; Sad; Tender; Fearful; Angry	110	Unfamiliar	116 raters from Finland	9-point scale	Positive/Negative valence; Positive/Negative energy; Negative energy; Positive/Negative tension; Happy; Sad; Tender; Fearful; Angry
Hill and Palmer, 2010	Affective response to a set of new musical stimuli	Self-composed using to major and minor mode	150 s,	Single-line melody in a piano keyboard timbre	**Emotion** Semantic Differential Feeling and Mood Scale (SDFMS): Elated—Depressed; Relaxed–Anxious; Confident–Unsure; Energetic–Fatigued; Good-natured–Grouchy	82	Novel	50 raters from the USA	5-point scale	Happy; Sad
Vieillard et al., 2008	Happy, sad, scary and peaceful musical excerpts for research on emotions	Self-composed using major and minor model	12.4 s (Range: 9.2–16.4 s)	Single-line melody in a piano keyboard timbre	**Emotion** Discrete model: Happy; Sad; Scary; Peaceful	56	Novel	59 raters from Canada	10-point scale	Happy; Sad; Scary; Peaceful

The present study aims to establish an affective dataset for Chinese traditional instrumental music, laying a solid foundation for cross-cultural studies on perception and emotional processing. To ensure ecological validity, our music excerpts all came from naturalistic music (studio recordings of music pieces instead of electronically synthesized ones). Both the dimensional and discrete emotion models were used to evaluate the music excerpts. A two-dimensional (valence–arousal) model was employed to address dimensional emotions to enable an accurate comparison with previous studies on Western music. The seven-factor discrete emotion (anger, gentleness, happiness, peacefulness, sadness, solemnness and transcendence) model, a domain-specific discrete emotion model constructed by Shi (2015) for addressing Chinese traditional music, was employed to evaluate the discrete emotions of the excerpts in the dataset.

The methodology for constructing the seven-factor discrete emotion model (Shi, 2015) resembled that of Zentner et al. (2008), encompassing three essential steps: compiling emotion terms related to music, conducting exploratory factor analysis to uncover the underlying structure, and performing confirmatory factor analysis to corroborate the findings (Shi, 2015). The emotions of anger, happiness, and sadness in the model are basic emotions (Ekman, 1992) that are extensively employed in emotion studies (Laukka et al., 2013; Lepping et al., 2016). The categories of gentleness and peacefulness are often used in music emotion research to represent neutral emotions, and these two emotion dimensions can be found in the GEMS (Zentner et al., 2008). Solemnness and transcendence are considered important expressive qualities of music and have been used as aesthetic emotion in several studies (Gabrielsson & Lindström, 1995; Brattico & Pearce, 2013; Akkermans et al., 2018; Vuilleumier & Trost, 2015; Zentner et al., 2008). This type of non-basic, complex emotion can be perceived by intrinsic and associative coding (Juslin, 2013b). It is more influenced by cultural factors (Matsumoto & Hwang, 2012) and associated with human social functions (Stellar et al., 2017).

The primary objective of the present study is to provide a novel dataset and validate its effectiveness. Concurrently, we aim to carry out a comparative analysis between dimensional and discrete models for emotional representation, to analyze the relationships within and across the two models, and to address the influence of musical familiarity on music emotion perception. We hypothesize that (1) intense typical emotions can be effectively described using either the dimensional or discrete emotion model, and moderate typical emotions can be better described with the dimensional than the discrete emotion model; (2) the emotions in the same valence–arousal space are more strongly correlated with each other; and (3) familiarity enhances emotion perception.

## Methods

### Stimuli development

In order to obtain a large sample of Chinese traditional instrumental music excerpts, we organized an expert panel consisting of musicians who had studied a musical instrument for more than 10 years and graduate students in psychology to develop the stimuli in the dataset.

Initially, the panel selected, based on their expertise, diverse Chinese traditional instrumental music, either well known or less familiar. A total of 145 ensemble performance records were sampled from internet archives. All the music pieces were performed by instrumental ensembles utilizing traditional Chinese “bayin” instruments, spanning from the Qin dynasty to the twentieth century and expressing a wide range of emotions.

Subsequently, using Audition CC software, each music piece was edited into one to four excerpts, each lasting 10 seconds, and beginning at musical phrase boundaries housing the core melodies. The duration of the excerpts is 10 seconds, and is used for scientific purposes, constituting fair use. The attributes of the excerpts were considered during the process of editing to ensure that the music elements like notes, rhythm, or timbre did not vary too much, avoiding a change in music emotion in a given excerpt.

Lastly, the music excerpts underwent batch processing, ensuring uniformity in formatting. A symmetric 1000-ms fade was applied at the beginning and at the end of each excerpt to make the excerpt sound more natural. The average sound intensity was consistent across all excerpts. Each excerpt was sampled at a rate of 44 kHz, and saved in MP3 format at a bit-rate of 192 kbps. Ultimately, a total of 280 excerpts were produced, of which seven were used for practice experiment and 273 for formal experiment.

### Stimuli validation

#### Participants

The Ryerson Audio-Visual Database of Emotional Speech and Song created by Livingstone and Russo (2018) and the emotional music database created by Vieillard et al. (2008) both used a small number of participants (≤ 20) for ratings. Belfi (2019) reported a fairly large individual difference in rating musical stimuli and used a relatively large number of participants (≥ 50) for rating each stimulus (Belfi & Kacirek, 2021). Therefore, we chose to use more than 50 participants for rating of each musical excerpt.

A total of 168 students (84 female; 84 male) from Hangzhou Normal University were recruited and involved in the present study (*M*_years_ = 22.36, *SD*_years_ = 2.14, range = [18, 33]). All the participants had normal hearing, had received no professional musical training (aside from the requirements of the general music curriculum in school), and could listen to/enjoy music in daily life. Each participant signed an informed consent and received monetary compensation for participation. The study was approved by the Ethics Committee of Hangzhou Normal University.

#### Procedures

The experimental procedures were programmed using E-Prime 3.0 software (Psychology Software Tools, Pittsburgh, PA, USA) and were shown on a 21.5-inch Dell monitor screen (1920 ×1080 pixels). Participants were required to wear headphones (SENNHEISER HD 200 PRO) for the music presentation. Participants were allowed to adjust the sound intensity of the music to a comfortable level before the experiment.

The 273 excerpts were divided into three lists, each containing 91 excerpts. To balance the sequential positions of the seven discrete emotion categories, we created four versions of the presentation, thus producing a total of 12 subroutines (3 lists of excerpts × 4 presentation versions). Each participant was randomly assigned to one of the 12 subroutines. To avoid fatigue, the excerpts in each list were separated into two blocks for presentation, one containing 45 excerpts and the other 46 excerpts. Participants could rest after completing the first block task. The sequential position of the presentation was randomized for each excerpt.

**Fig. 1:**
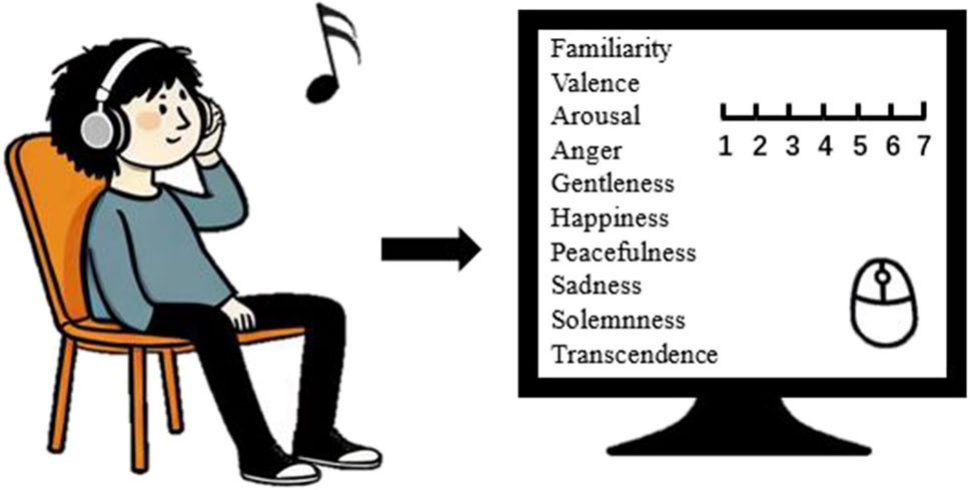
Schematic showing excerpt rating

Participants were required to rate ten variables on a seven-point Likert scale for each excerpt: familiarity, dimensional emotions (valence and arousal), and discrete emotions (anger, gentleness, happiness, peacefulness, sadness, solemnness, and transcendence) **(**Fig. 1**)**.

For ***familiarity***, participants were instructed: [*Please rate on the scale of familiarity level of the music from 1 to 7, where 1 means “not at all familiar,” 2 “slightly familiar,” 3 “somewhat familiar,” 4 “moderately familiar,” 5 “very familiar,” 6 “strongly familiar,” and 7 “extremely familiar”].*

For ***valence***, participants were instructed: [*Please rate on the scale of valence level of the music, from 1 (“extremely negative”) to 7 (“extremely positive”), of which 4 represents “neutral”)*]. For ***arousal***, participants were instructed: *[Please rate on the scale the arousal level of the music, from 1 (“not at all aroused”) to 7 (“extremely aroused”), of which 4 represents “moderately aroused”*].

For discrete emotions *(****anger, gentleness, happiness, peacefulness, sadness, solemnness, and transcendence****)*, participants were instructed: [*Please rate on the scale of each emotion based on the intensity you experience while listening to the music, from 1 (“nonexistent”) to 7 (“extremely intense”), of which 4 represents “moderately intense”*]. Participants were required to select at least one among the seven discrete emotions for rating. Unselected emotions were arbitrarily rated as a value of 1 (“*nonexistent")*.

The present work focused primarily on perceived emotions. Perceived emotion refers to an emotion expressed by music that a listener feels. For each trial, an excerpt was played once on the first screen where participants were required to assess the familiarity and dimensional emotion categories, followed by a second screen where the excerpts were played once again and participants were required to rate the category of discrete emotion for the excerpt. After completing all of the ratings for one excerpt, participants clicked the “NEXT” button to initiate the next trial. It took about 1.5 hours for each participant to complete a whole rating task.

### Data analysis

**Fig. 2:**
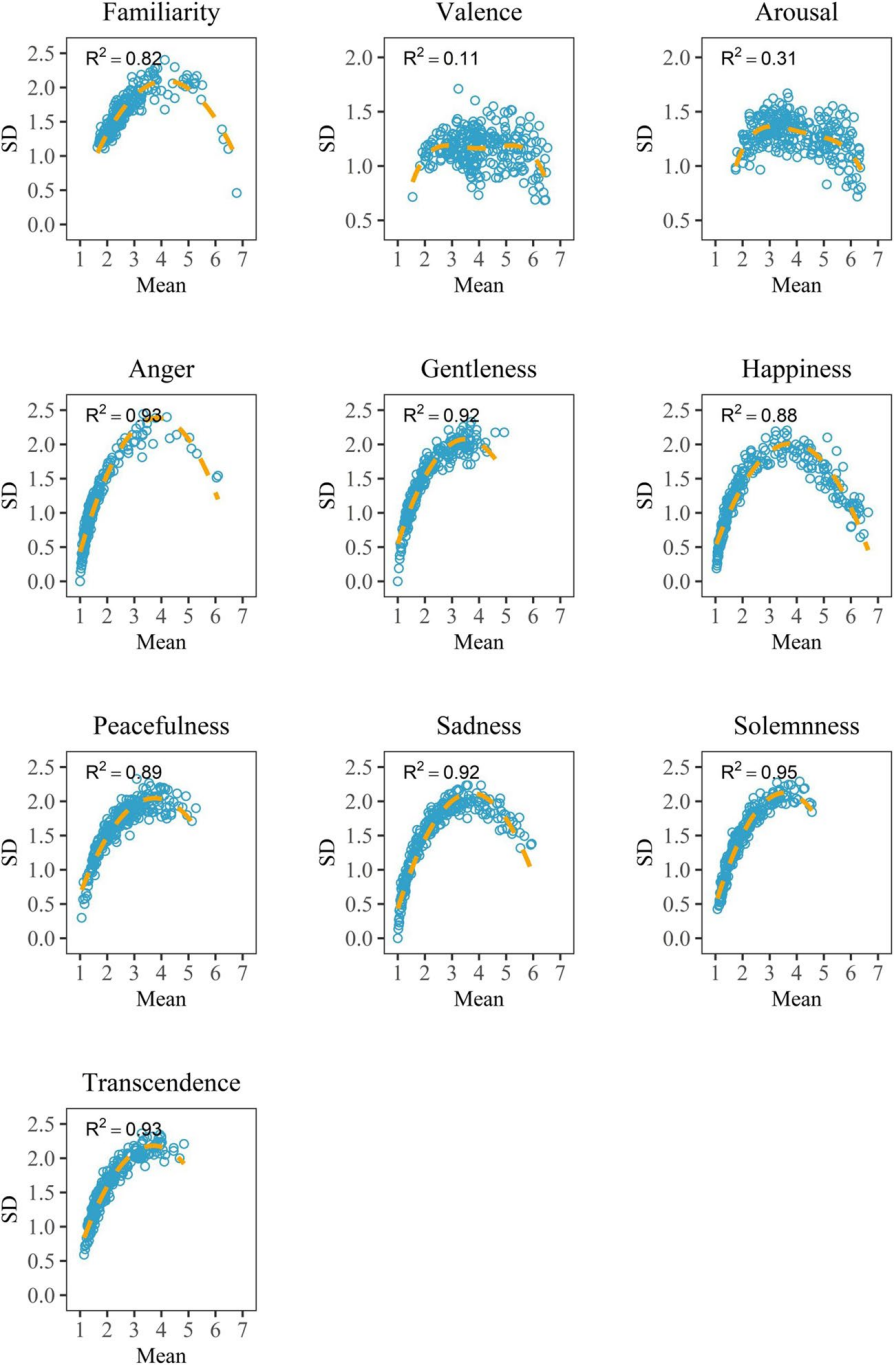
Plots of *SD* against the mean value for the ten variables

A comprehensive analysis overview is presented here. Initially, we analyzed the reliability and distribution of the data. The intra-class correlation coefficient (ICC) was used to measure the consistency between the raters among all the variables (see Table 2). The relationship between the means and standard deviations among all the variables was explored to address the rating distribution for each variable (see Fig. 2). Pearson correlation analysis was performed to address the relationships between dimensional emotions and between discrete emotions (see Fig. 5).

**Table 2:** Inter-rater reliability for the ten variables

Variable	ICC type	ICC value	95% Confidence interval	
			Lower bound	Upper bound
Familiarity	Single (1,1)	0.12	0.085	0.169
	Average (1,k)	0.97	0.963	0.982
Valence	Single (1,1)	0.02	0.015	0.035
	Average (1,k)	0.86	0.811	0.908
Arousal	Single (1,1)	0.03	0.022	0.048
	Average (1,k)	0.90	0.860	0.933
Anger	Single (1,1)	0.04	0.026	0.056
	Average (1,k)	0.91	0.879	0.943
Gentleness	Single (1,1)	0.06	0.043	0.091
	Average (1,k)	0.95	0.926	0.965
Happiness	Single (1,1)	0.02	0.013	0.030
	Average (1,k)	0.84	0.785	0.894
Peacefulness	Single (1,1)	0.05	0.035	0.075
	Average (1,k)	0.94	0.910	0.957
Sadness	Single (1,1)	0.04	0.025	0.054
	Average (1,k)	0.91	0.876	0.941
Solemnness	Single (1,1)	0.05	0.033	0.072
	Average (1,k)	0.93	0.905	0.955
Transcendence	Single (1,1)	0.06	0.042	0.089
	Average (1,k)	0.95	0.924	0.964

To determine the familiarity label of each excerpt, we calculated the mean rating for the familiarity variable. An excerpt with a mean rating larger than 3 (“*somewhat familiar”*) was labeled as familiar, and that smaller than 3 as unfamiliar. To determine the discrete emotion label for each excerpt, we computed the mean rating of each discrete emotion variable and assigned the highest rating among the seven categories as the discrete emotion label.

To validate the effectiveness of music label categorization and to illustrate differences in music emotion attributes across various classifications, the following statistical analyses were conducted. For each analysis, the dependent variable was the mean rating of the corresponding dimension. The paired-samples* t*-test was used to examine differences in familiarity ratings between familiar and unfamiliar music. To validate the effectiveness of the labeled emotion in each discrete music emotion category, repeated-measures ANOVAs were performed with the seven discrete emotions (anger, gentleness, happiness, peacefulness, sadness, solemnness, and transcendence) as the within-subject factor. Post hoc tests were carried out to compare the rating of a labeled emotion with that of each of the other emotions. To explore distinct emotional properties (valence, arousal, and intensity) within the seven emotion categories of music, repeated-measures ANOVAs were performed with the seven discrete music emotion categories (anger, gentleness, happiness, peacefulness, sadness, solemnness, and transcendence) as the within-subject factor. To address whether familiarity influences emotion perception (valence, arousal, and labeled emotion intensity), the paired-samples *t*-test or Wilcoxon–Mann–Whitney test was conducted, with familiarity (familiar and unfamiliar) as the within-subject factor.

A data-driven analysis using k-means clustering was performed to classify emotion categories. The following statistical analyses were used to address differences in emotional attributes across different k-cluster categories (Clusters 1–4), with mean ratings as dependent variable. To explore the distinct emotion properties (valence, arousal, and labeled emotion intensity) within the four k-cluster categories, repeated-measures ANOVAs were performed with k-cluster category as the within-subject factor. To investigate the discrete emotion properties in each cluster, repeated-measures ANOVAs were conducted with the seven discrete emotions (anger, gentleness, happiness, peacefulness, sadness, solemnness, and transcendence) as the within-subject factor. Finally, the potential impact of sex on ratings was assessed using an independent-samples *t*-test across ten variables.

The assumptions of homogeneity and sphericity were assessed using Mauchly's test within the repeated-measures ANOVA. The Greenhouse–Geisser correction was used if the model assumption was not met. Bonferroni correction was applied for pairwise comparisons. An alpha level of 0.05 was used for all statistical tests.

## Results

### Inter-rater reliability

Using the ICC function from the “IRR” package in R (4.2.1) (Hallgren, 2012), the inter-rater reliability across the ten variables was calculated. ICCs were calculated for both single and average ratings (see Table 2). The result shows that the ten variables (familiarity, valence, arousal, anger, gentleness, happiness, peacefulness, sadness, solemnness, and transcendence) have low agreement for single measurements but high agreement for average measurements (Koo & Li, 2016).

### Distributions of ratings

The mean rating and the corresponding standard deviation (*SD*) were calculated for each excerpt in the ten variables. Figure 2 shows the plotting of *SD* against the mean value. As we can see, there exist three types of distributions: (1) M-shaped distribution for the valence and arousal variables, (2) inverted U-shaped distribution for the familiarity, anger, happiness, and sadness variables, and (3) half-inverted U-shaped distribution for the gentleness, peacefulness, solemnness, and transcendence variables. A quartic regression demonstrated the best fit to the data, as indicated by the fitted curves and the *R*-square value (Fig. 2).

### Correlations between each emotion model

We explored the relationships between the dimensional emotion variable by calculating Pearson’s correlations between valence and arousal. Those two variables were significantly positively correlated (*r* = 0.77, *p* < .001).

**Fig. 3:**
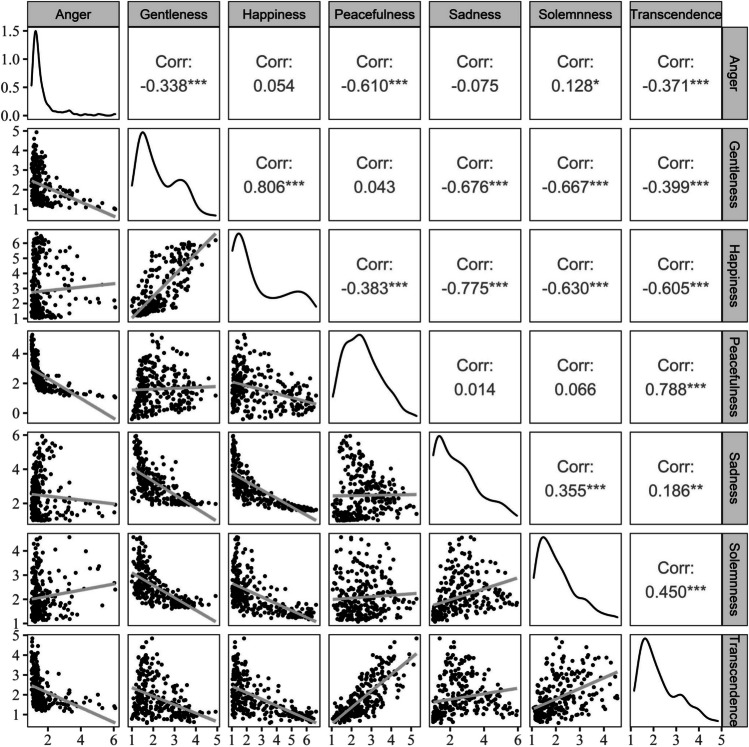
Pairwise correlations between the seven discrete emotions

We investigated the relationships among the discrete emotion variables by calculating Pearson’s correlations between each pair of variables. Figure 3 shows a graphical depiction of these correlations.

### Categorization of emotions

The average ratings were calculated for each of the ten variables. The classification of familiarity was based on the ratings averaged across participants. Music excerpts rated 3 or higher were labeled as familiar and those below 3 as unfamiliar. In this way, we obtained a total of 67 familiar excerpts (*M*_familiar_ = 4.05, *SD*_familiar_ = 0.97, range = [3, 6.78]) and 206 unfamiliar excerpts (*M*_*un*familiar_ = 2.32, *SD*_*un*familiar_ = 0.31, range = [1.64, 2.96]). A significant difference was detected between the familiar and unfamiliar excerpts in familiarity rating (*t*(271) = 22.43, *p* < .001, Cohen’s *d =* 3.15, 95% CI = [2.77, 3.54]). The discrete emotion label was determined by the highest rating among anger, gentleness, happiness, peacefulness, sadness, solemnness, and transcendence. For example, an excerpt was labeled as “happiness” if the “happiness” dimension had the highest mean rating score among the seven dimensions, and an excerpt was labeled as “sadness” if the “sadness” dimension had the highest mean rating score among the seven dimensions, and so on. In this way, a total of 12 music excerpts were categorized as anger, 3 as gentleness, 95 as happiness, 52 as peacefulness, 71 as sadness, 21 as solemnness, and 19 as transcendence. Table 3 shows the ratings for familiarity, valence, arousal, and labeled-emotion intensity for each discrete category under unfamiliar and familiar conditions. The excerpts in the gentleness, solemnness, and transcendence categories were all rated as unfamiliar, and therefore only four subcategories (anger, happiness, peacefulness, and sadness) contained familiar excerpts.

**Table 3:** Ratings of familiarity, valence, arousal, and labeled-emotion intensity for the discrete emotional music category under unfamiliar and familiar condition

Category	Condition	*N*	Familiarity		Valence		Arousal		Labeled-emotion intensity	
			Mean	*SD*	Mean	*SD*	Mean	*SD*	Mean	*SD*
Anger	*Total*	*12*	*3.34*	*0.21*	*4.08*	*0.21*	*5.57*	*0.28*	*4.52*	*1.04*
	Unfamiliar	10	2.30	0.17	4.00	0.18	5.51	0.31	4.50	1.23
	Familiar	2	4.39	0.38	4.51	0.35	5.91	0.02	4.62	0.35
Gentleness	Unfamiliar	3	2.56	0.31	4.40	0.11	3.92	0.01	3.38	0.11
Happiness	*Total*	*95*	*3.20*	*0.06*	*5.33*	*0.44*	*5.19*	*0.46*	*4.94*	*1.07*
	Unfamiliar	48	2.44	0.08	4.97	0.26	4.88	0.34	4.42	0.79
	Familiar	47	3.95	0.08	5.71	0.36	5.50	0.39	5.48	0.79
Peacefulness	*Total*	*52*	*3.21*	*0.09*	*3.86*	*0.18*	*2.92*	*0.36*	*3.83*	*0.37*
	Unfamiliar	39	2.31	0.09	3.79	0.15	2.86	0.38	3.81	0.40
	Familiar	13	4.11	0.15	4.06	0.22	3.11	0.30	3.88	0.29
Sadness	*Total*	*71*	*3.48*	*0.13*	*2.82*	*0.35*	*3.26*	*0.38*	*4.15*	*0.77*
	Unfamiliar	66	2.25	0.07	2.79	0.34	3.26	0.40	4.15	0.79
	Familiar	5	4.71	0.24	3.15	0.49	3.33	0.13	4.14	0.65
Solemnness	Unfamiliar	21	2.40	0.12	3.49	0.12	3.37	0.56	3.75	0.34
Transcendence	Unfamiliar	19	2.23	0.12	3.39	0.07	2.67	0.32	3.73	0.26

**Fig. 4:**
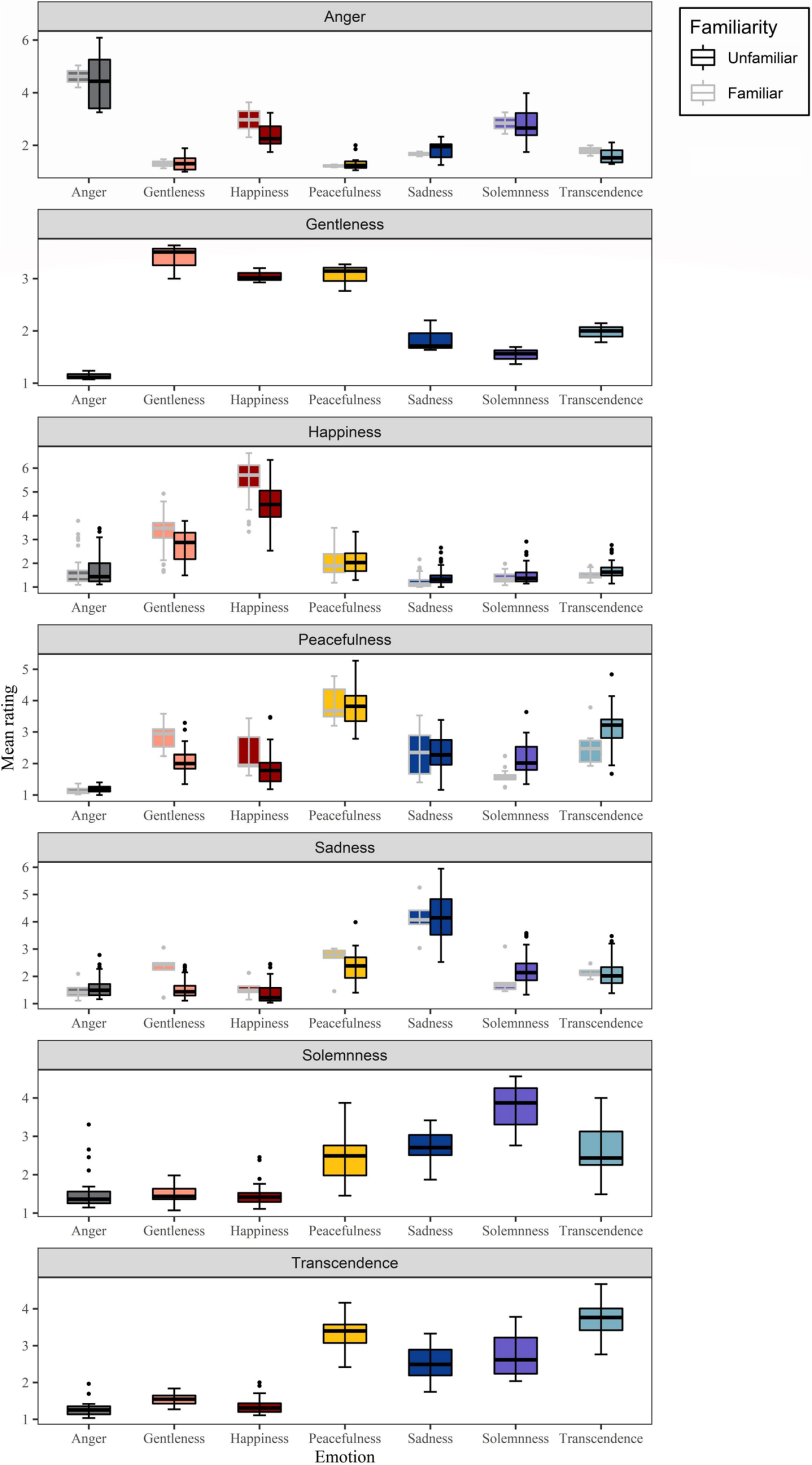
Mean ratings and 95% confidence intervals for the discrete emotion categories

Repeated-measures ANOVA was performed to examine the validity of labeled emotion. We conducted the analysis for ANOVA using the “avo” function in R. The main effect was significant in all groups (all *p* < .001) (for details see Table 4). Post hoc analysis revealed that the rating for labeled emotions was significantly higher than those for non-labeled emotions in the anger, happiness, peacefulness, sadness, and solemnness categories (all *p* < .001; Fig. 4). In the gentleness category, the rating for gentleness was significantly higher than those for the non-labeled emotions except for happiness and peacefulness, and in the transcendence category, the rating for transcendence was significantly higher than those for the non-labeled emotions except for peacefulness (Fig. 4**)**.

**Table 4:** ANOVA results for each discrete emotional music category

Discrete emotion categories	*F *value	Post hoc analysis
Anger	8.95 ***	*G, H, P, Sa, S, T*
Gentleness	43.56 ***	*A, Sa, S, T*
Happiness	350.41***	*A, G, P, Sa, S, T*
Peacefulness	38.56 ***	*A, H, G, Sa, S, T*
Sadness	14.09 ***	*A, G, H, Sa, S, T*
Solemnness	55.67 ***	*A, G, H, P, Sa, T*
Transcendence	109.05 ***	*A, G, H, Sa, S*

A = Anger, G = Gentleness, H = Happiness, P = Peacefulness, Sa = Sadness, S = Solemnness, T = Transcendence. Post hoc tests display labeled emotions that differ in means. *p < .05; **p < .01; ***p < .001

#### Comparison of valence, arousal, and intensity ratings

**Fig. 5:**
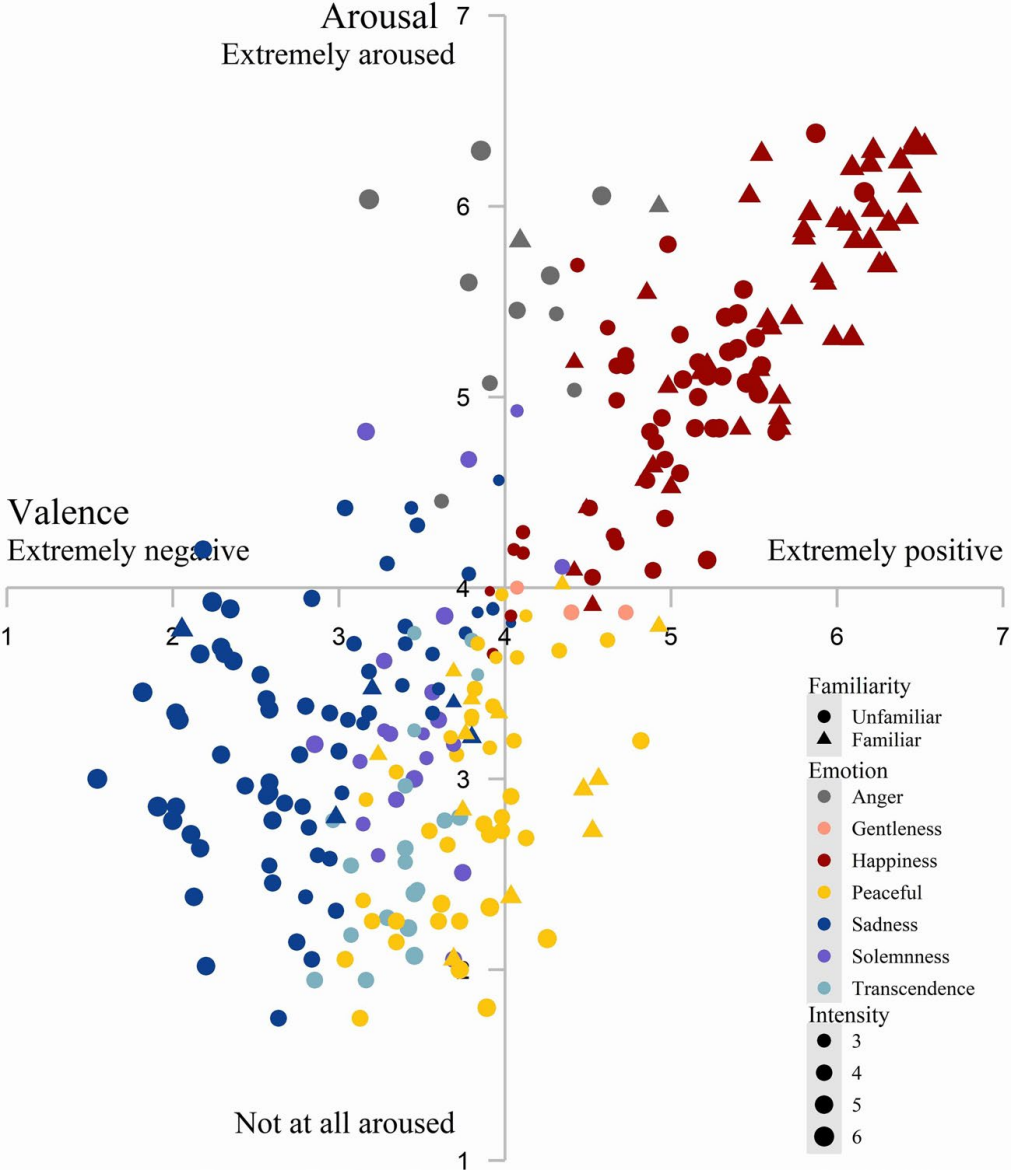
Distributions of the ratings for arousal and valence and labeled-emotion intensity averaged across participants for each excerpt under familiar and unfamiliar conditions

Figure 5 shows the distributions of the ratings for arousal and valence and the labeled-emotion intensity averaged across participants for each excerpt under familiar and unfamiliar conditions. Repeated-measures ANOVA was performed to compare the ratings of valence, arousal, or labeled-emotion intensity among the seven discrete emotions.

For the valence rating, the main effect of discrete emotion type was significant (*F*(6, 266) = 152.70, *p* < .001, *η*^2^_p_ = 0.77, 90% CI = [0.73, 0.80]). Post hoc (Tukey) test showed: happiness^a^ (*M* = 5.33, *SD* = 0.44) > gentleness^b^ (*M* = 4.40, *SD* = 0.11) > anger^bc^ (*M* = 4.08, *SD* = 0.21) > peacefulness^bcd^ (*M* = 3.86, *SD* = 0.18) > solemnness^cd^ (*M* = 3.49, *SD* = 0.12) > transcendence^de^ (*M* = 3.39, *SD* = 0.07) > sadness^e^ (*M* = 2.82, *SD* = 0.35) (a > b > c > d > e).

For the arousal rating, the main effect of discrete emotion type was significant (*F*(6, 266) = 124.07, *p* < .001, *η*^2^_p_ = 0.74, 90% CI = [0.69, 0.76]). Post hoc test revealed: anger^a^ (*M* = 5.57, *SD* = 0.28) > happiness^a^ (*M* = 5.19, *SD* = 0.46) > gentleness^b^ (*M* = 3.92, *SD* = 0.01) > solemnness^bc^ (*M* = 3.37, *SD* = 0.56) > sadness^bc^ (*M* = 3.26, *SD* = 0.38) > peacefulness^c^ (*M* = 2.92, *SD* = 0.36) > transcendence^c^ (*M* = 2.67, *SD* = 0.32) (a > b > c).

For labeled-emotion intensity, the main effect of discrete emotion type was also significant (*F*(6, 266) = 15.39, *p* < .001, *η*^2^_p_ = 0.26, 90% CI = [0.17, 0.31]). Post hoc test demonstrated: happiness^a^ (*M* = 4.94, *SD* = 1.07) > anger^ab^ (*M* = 4.52, *SD* = 1.04) > sadness^abc^ (*M* = 4.115, *SD* = 0.77) > peacefulness^bc^ (*M* = 3.83, *SD* = 0.337) > solemnness^bc^ (*M* = 3.75, *SD* = 0.34) > transcendence^bc^ (*M* = 3.73, *SD* = 0.26) > gentleness^c^ (*M* = 3.38, *SD* = 0.11) (a>b>c).

In order to determine whether familiarity influences emotion perception, we compared the valence, arousal, or labeled-emotion intensity between familiar and unfamiliar conditions. Independent-samples *t*-tests were used for the happiness and peacefulness categories, and a significant difference was detected for the happiness category. Familiar excerpts were rated as more positive (*M*_v_familiar_ = 5.71, *SD*_v_familiar_ = 0.60; *M*_v_unfamiliar_ = 4.97, *SD*_v_unfamiliar_ = 0.51, *t*(93) = 6.52, *p* < .001, Cohen’s *d* = 0.67, 95% CI = [0.45, 0.90]), more arousing (*M*_a_familiar_ = 5.50, *SD*_a_familiar_ = 0.63; *M*_a_unfamiliar_ = 4.88, *SD*_a_unfamiliar_ = 0.58, *t*(93) = 5.05, *p* < .001, Cohen’s *d* = 0.52, 95% CI = [0.31, 0.74]), and more intense (*M*_i_familiar_ = 5.48, *SD*_i_familiar_ = 0.89; *M*_i_unfamilia r_ = 4.42, *SD*_i_unfamiliar_ = 0.89, *t*(93) = 5.80, *p* < .001, Cohen’s *d* = 0.60, 95% CI = [0.38, 0.82]) relative to the unfamiliar ones. Thus, familiarity enhances the perception of happiness emotion. The Wilcoxon–Mann–Whitney test was used for anger and sadness categories, and no difference was detected between familiar and unfamiliar conditions. We conducted Pearson correlation analysis in which familiarity was used as a continuous variable to assess its impact on emotion perception (see Supplementary file).

#### Cluster analysis of discrete emotions

As seen in the *Emotion categorization* section, the highest discrete emotion ratings were used to group the excerpts into seven discrete emotion categories. It was unclear whether these categories differed systematically. To address this issue, here we conducted a k-means cluster analysis to classify the excerpts based on their normative ratings. We aimed to determine whether we could “group” excerpts based on their normative assessments, and if so, whether these groups would be systematically mapped onto the discrete emotion categories.

**Fig. 6:**
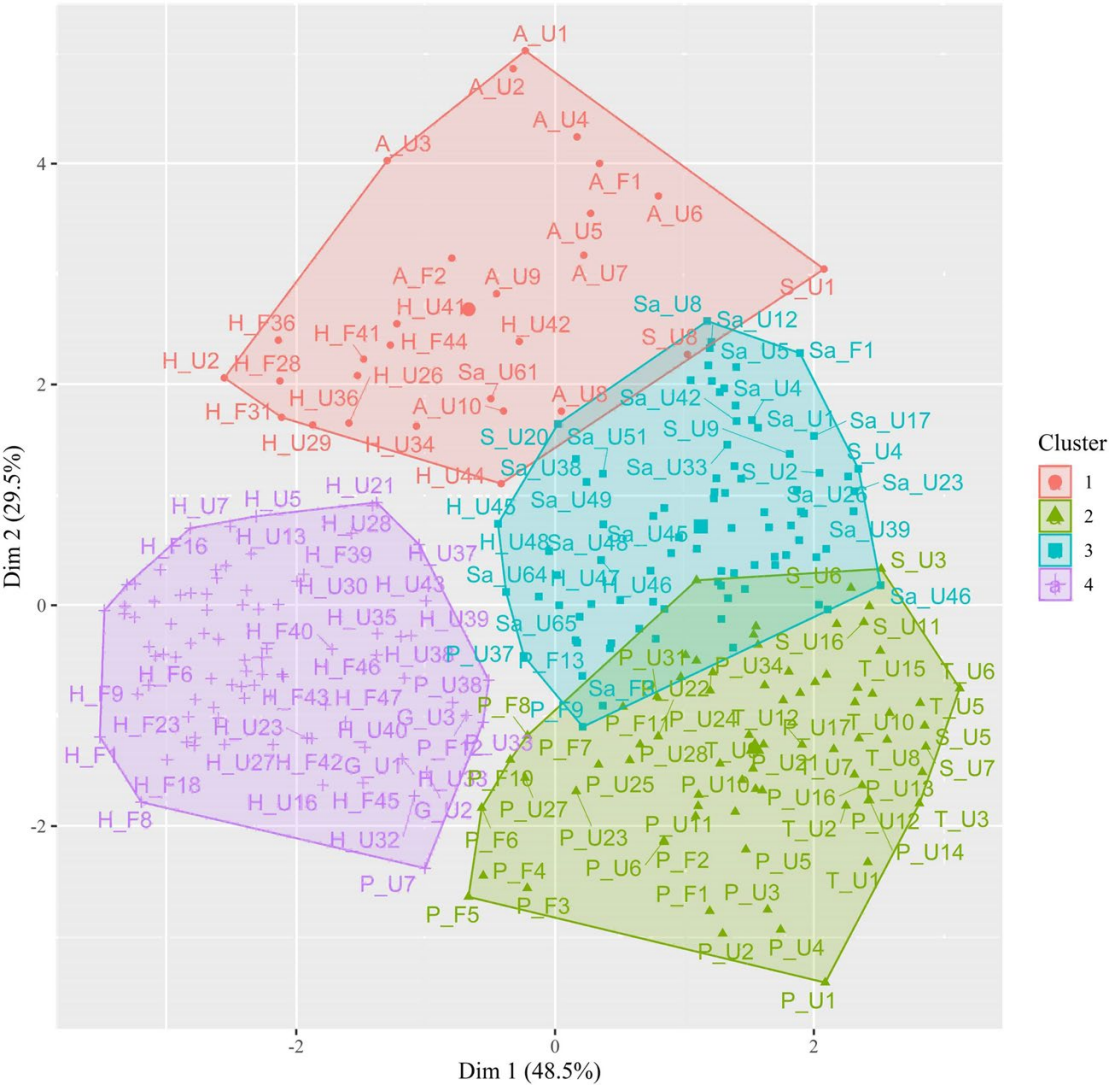
Cluster visualization. Principal component analysis (PCA) was performed to visualize the 7-dimensional data using the two principal components (Dim 1 and 2), capturing 78% (Dim1 48.5% +Dim2 29.5%) of variance in the entire rating dataset. Clustering could be clearly seen within clusters and separating between clusters. The character labels represent discrete emotion categories, familiarity types and ranking (descending order) of labeled-emotion intensity (A=Anger, G=Gentleness, H=Happiness, P=Peacefulness, Sa=Sadness, S=Solemnness, T=Transcendence; U=Unfamiliar, F=Familiar). To maintain readability, only a portion of excerpt labels are included

First, we determined the ideal number of clusters based on normative rating data. To do so, we converted the ratings to *z*-scores. An analysis was run in R to determine the optimal number of clusters, using the NbClust function from the NbClust package (Charrad et al., 2014). The NbClust provided 30 indices for determining cluster numbers and proposed the best clustering scheme by varying combinations of cluster numbers, distance measures, and clustering methods. According to the majority rule, four cluster groups were determined as the optimal clustering (eight various indices proposed). Figure 6 shows a graphical depiction of these clusters.

**Fig. 7:**
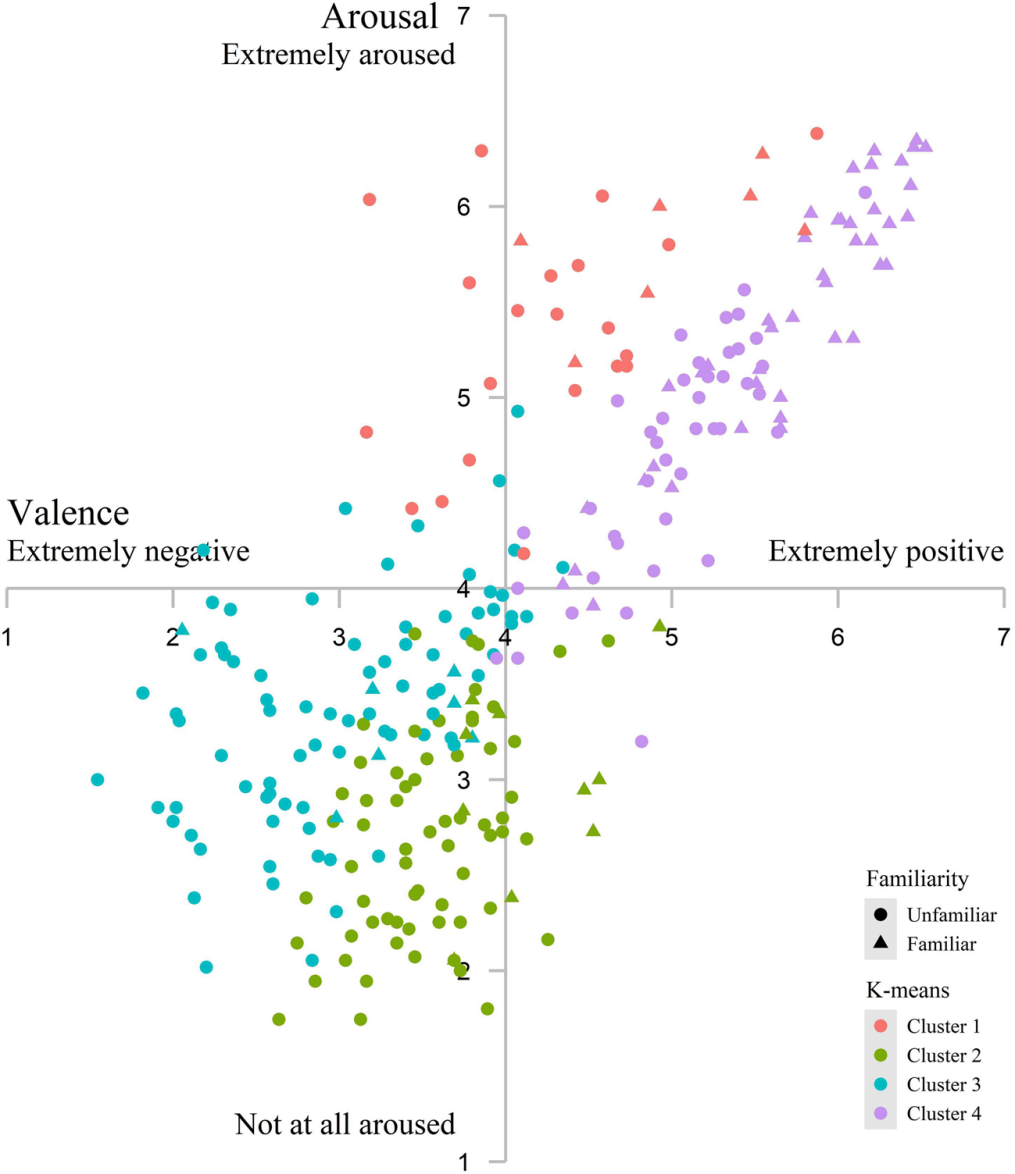
Ratings of valence and arousal for each excerpt grouped by familiarity and K-means

K-means clustering was carried out using the k-means function in R with 4 as the preset number of clusters, producing a total of 28 excerpts in Cluster 1, 74 in Cluster 2, 86 in Cluster 3, and 85 in Cluster 4. The average ratings for valence, arousal, and labeled-emotion intensity across participants for each cluster are shown in Table 5. Repeated-measures ANOVA was performed to compare the ratings of valence, arousal or labeled emotion intensity among the four clusters. The main effect of cluster was significant for the ratings of valence (*F*(3, 269) = 222.41, *p* < .001, *η*^2^_p_ = 0.71, 90% CI = [0.67, 0.74]) and arousal (*F*(3, 269) = 269.41, *p* < .001, *η*^2^_p_ = 0.75, 90% CI = [0.71, 0.88]). Post hoc test for valence showed: Cluster 4^a^ (*M* = 5.37, *SD* = 0.43) > Cluster 1^b^ (*M* = 4.42, *SD* = 0.52) > Cluster 2^c^ (*M* = 3.61, *SD* = 0.21)> Cluster 3^d^ (*M* = 3.02, *SD* = 0.46) ( a > b > c > d). Post hoc test for arousal showed: Cluster 1^a^ (*M* = 5.45, *SD* = 0.35) > Cluster 4^b^ (*M* = 5.08, *SD* = 0.54) > Cluster 3^c^ (*M* = 3.38, *SD* = 0.32) > Cluster 2^d^ (*M* = 2.73, *SD* = 0.29) (a > b > c > d). The main effect of Cluster was not significant for intensity (*F*(3, 269) = 0.20, *p* = 0.90). Figure 7 shows the ratings of valence and arousal for each excerpt grouped by familiarity and cluster.

**Table 5:** Average ratings for valence, arousal, and labeled-emotion intensity across participants for each cluster

K-means	*n*	Valence		Arousal		Labeled-emotion intensity	
		Mean	*SD*	Mean	*SD*	Mean	*SD*
Cluster 1	28	4.42	0.52	5.45	0.35	4.22	0.19
Cluster 2	74	3.61	0.21	2.73	0.29	4.34	0.12
Cluster 3	86	3.02	0.46	3.38	0.32	4.36	0.11
Cluster 4	85	5.37	0.43	5.08	0.54	4.27	0.11

For each of the clusters, we conducted repeated-measures ANOVA on the factor of discrete emotion. For Cluster 1, the main effect of discrete emotion type was significant (*F*(6, 189) = 23.11, *p* < .001, *η*^2^_p_ = 0.42, 90% CI = [0.32, 0.48]). Post hoc test showed: anger^a^ (*M* = 3.67, *SD* = 1.03) > happiness^a^ (*M* = 3.28, *SD* = 1.24) > solemnness^b^ (*M* = 2.33, *SD* = 0.94) > sadness^bc^(*M* = 1.77, *SD* = 0.52) > transcendence^c^ (*M* = 1.58, *SD* = 0.29) > gentleness^c^ (*M* = 1.57, *SD* = 0.34) > peacefulness^c^ (*M* = 1.39, *SD* = 0.24) (a > b > c).

For Cluster 2, the main effect of discrete emotion type was significant (*F*(6, 511) = 177.83, *p* < .001, *η*^2^_p_ = 0.68, 90% CI = [0.64, 0.70]). Post hoc test indicated: peacefulness^a^ (*M* = 3.67, *SD* = 0.62) > transcendence^b^ (*M* = 3.33, *SD* = 0.59) > sadness^c^ (*M* = 2.47, *SD* = 0.62) > solemnness^c^ (*M* = 2.45, *SD* = 0.77) > gentleness^d^ (*M* = 1.92, *SD* = 0.53) > happiness^e^ (*M* = 1.64, *SD* = 0.53) > anger^f^ (*M* = 1.21, *SD* = 0.15) (a > b > c > d > e > f).

For Cluster 3, the main effect of discrete emotion type was significant (*F*(6, 595) = 155.13, *p* < .001, *η*^2^_p_ = 0.61, 90% CI = [0.57, 0.64]). Post hoc test exhibited: sadness^a^ (*M* = 3.83, *SD* = 1.03) > solemnness^b^ (*M* = 2.37, *SD* = 0.71) > peacefulness^bc^ (*M* = 2.32, *SD* = 0.47) > transcendence^c^ (*M* = 2.10, *SD* = 0.39) > gentleness^d^ (*M* = 1.64, *SD* = 0.44) > anger^d^ (*M* = 1.58, *SD* = 0.35)> happiness^d^ (*M* = 1.53, *SD* = 0.48) (a > b > c > d).

For Cluster 4, the main effect of discrete emotion type was significant (*F*(6, 588) = 543.00, *p* < .001, *η*^2^_p_ = 0.85, 90% CI = [0.83, 0.86]). Post hoc test displayed: happiness^a^ (*M* = 4.98, *SD* = 1.06) > gentleness^b^ (*M* = 3.30, *SD* = 0.52) > peacefulness^c^ (*M* = 2.25, *SD* = 0.58) > transcendence^d^ (*M* = 1.62, *SD* = 0.29) > anger^de^ (*M* = 1.42, *SD* = 0.29) > solemnness^e^ (*M* = 1.39, *SD* = 0.22) > sadness^e^ (*M* = 1.31, *SD* = 0.32) (a > b > c > d > e).

**Fig. 8:**
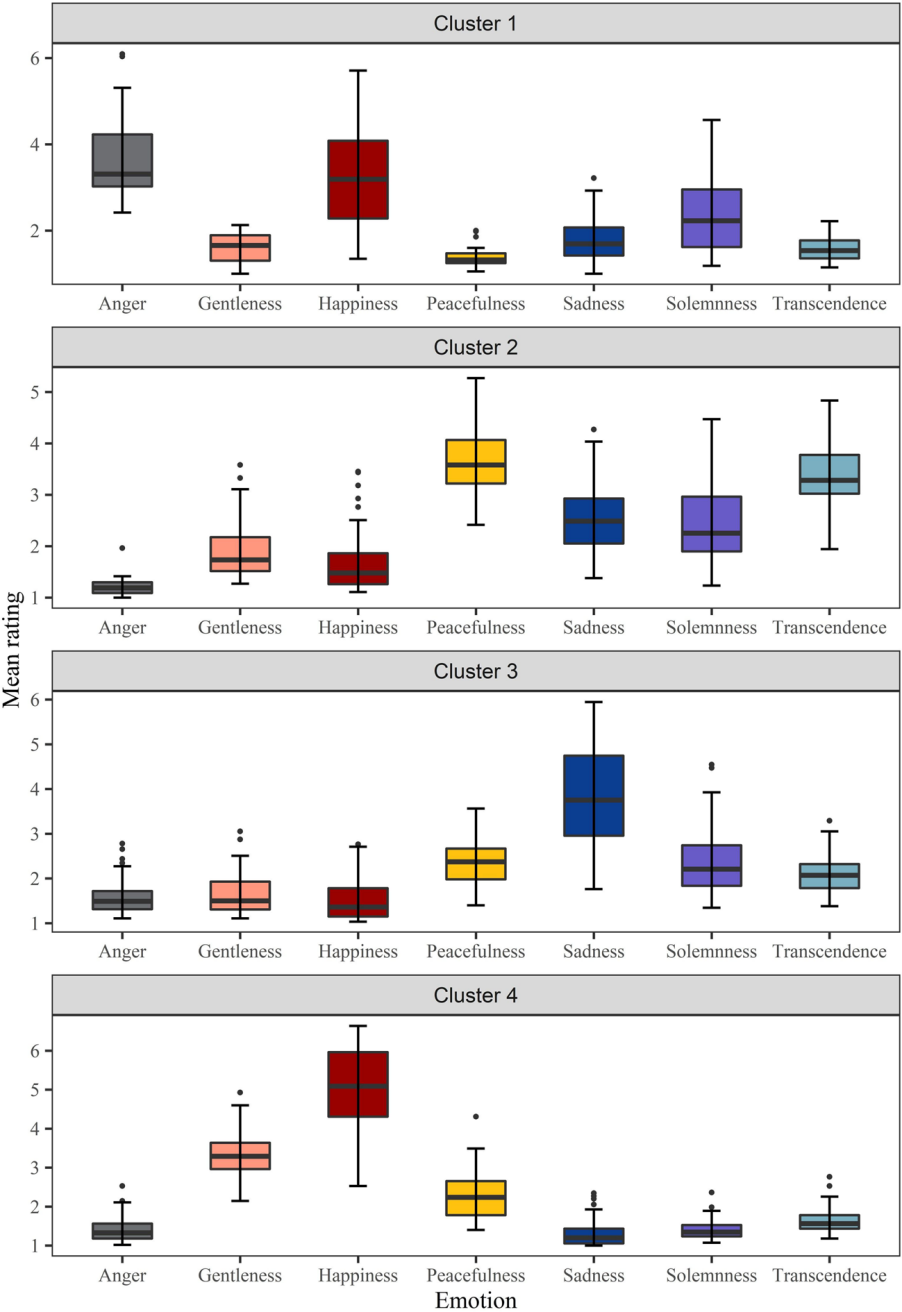
Mean ratings and 95% confidence intervals of the seven discrete emotions in each cluster

**Fig. 9:**
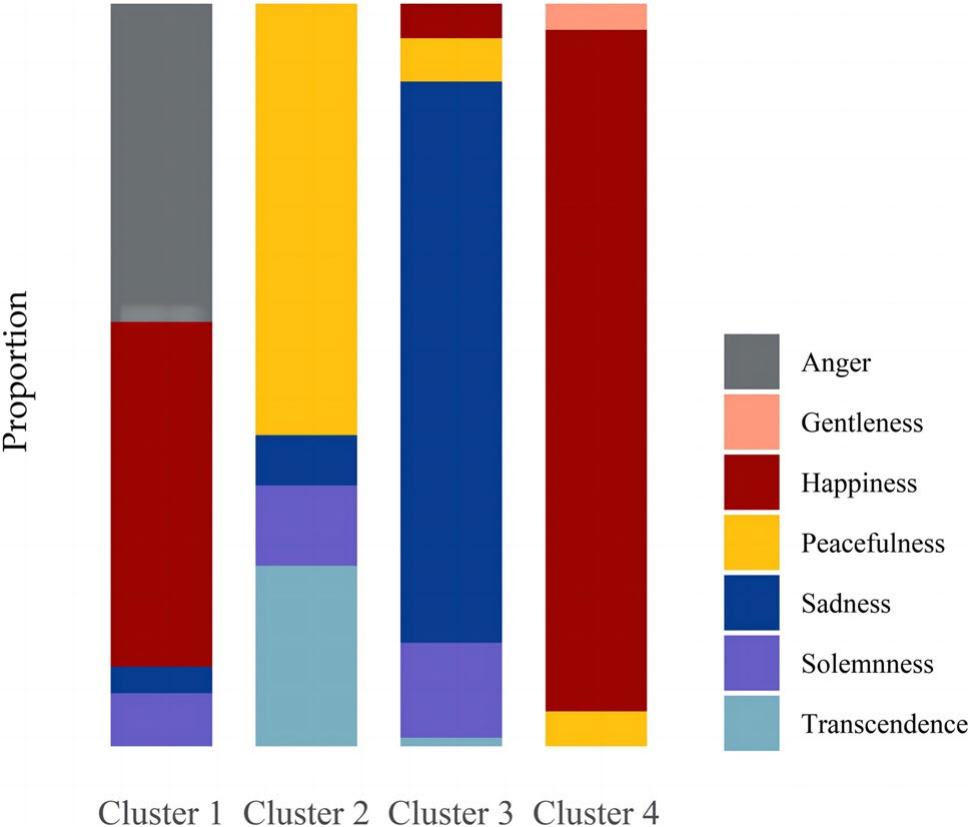
Proportions of the seven discrete emotion excerpts in each cluster

In summary, Cluster 1 consists of excerpts rated as more anger and happiness, Cluster 2 excerpts rated as more peacefulness and transcendence, Cluster 3 excerpts rated as more sadness, and Cluster 4 excerpts rated as more happiness. Figure 8 shows the mean ratings of the seven discrete emotions in each cluster, and Fig. 9 displays the proportional distributions of the excerpts in each cluster.

Finally, we analyzed the potential effects of the sex difference on the emotion ratings. Independent-samples *t*-tests revealed that participant sex had a significant effect only on the familiarity rating (*M*_male_ = 2.85, *SD*_male_ = 0.08; *M*_female_ = 2.64, *SD*_female_ = 0.05; *t*(547) = 2.64, *p* < .05, Cohen’s *d* = 0.39, 95% CI = [0.09, 0.68]).

## Discussion

The present study established a dataset of Chinese traditional instrumental music with rating data on familiarity, dimensional emotions, and discrete emotions. Three types of labels for the excerpts were obtained: familiarity, discrete emotion, and cluster. The majority of excerpts were rated as unfamiliar type, the dimensional emotion parameters were directly represented by numerical values, and the inter-rater reliability was excellent for average measurements.

The distribution of *SD* against the mean value of rating exhibited a similar pattern for the ten variables. Smaller variability in *SD* was found at the two ends of the mean scale (Fig. 2), indicating that high typical emotions expressed by music (e.g., extremely negative or extremely happy) can be identified more accurately. These high typical emotions can be described well with both dimensional and discrete emotion models, consistent with the conclusion by Eerola and Vuoskoski (2011). Meanwhile, increased variability was found at around the midpoint of the mean scale, suggesting that moderate typical emotions are identified ambiguously. Even so, the variables in the dimensional emotion model demonstrated smaller variability relative to those in the discrete emotion model, particularly at the midpoint of the mean scale, indicating that the dimensional emotion model is more reliable in describing moderate typical emotions than the discrete emotion model, consistent with the conclusion by Eerola and Vuoskoski (2011). For the familiarity variable, we assumed that non-musicians in the Chinese population shared almost the same experience of familiarity, which is highly familiar with some well-known traditional music but unfamiliar with other unknown music. The large variability in moderate familiar music excerpts was not in line with our expectations. This might be partly due to some famous music being too short to be recognized and some similar patterns for strange music eliciting the feeling of familiarity.

The dimensional emotions in the present study were highly correlated, consistent with previous studies concerning emotional music datasets (Belfi & Kacirek, 2021; Imbir & Gołąb, 2017), where a strong positive correlation was reported between valence and arousal. The distribution pattern of music excerpts within the valence–arousal quadrant in the present study was closely aligned with the previous dataset constructed by Koh et al. (2020). The positive valence excerpts were highly arousing whereas negative valence excerpts elicited a state of low arousal. This might be attributed to the intrinsic emotional properties conveyed by music.

The correlation patterns between the discrete emotions in the present study were similar to the original results of covariance analysis in the previous study constructing the discrete emotion model for Chinese traditional music (Shi, 2015). As seen in Fig. 5, “happiness” and “gentleness” are highly correlated with each other. The term “gentleness” corresponds to the term “tenderness” used in the previous study by Juslin (2001), in which positive emotions were linked with tenderness. As “transcendence” and “peacefulness” both induce relaxation, it makes sense that they were highly correlated with each other. “Peacefulness” and “transcendence” can also be seen in the GEMS as first-order dimensions and are listed under a second-order dimension called “sublimity” (Zentner et al., 2008). The difference is that while the Geneva “transcendence” emotion of Western music represents a spiritual and mystical feeling, the “transcendence” emotion of Chinese traditional music represents the supernatural and indifference (Shi, 2015).

Analyzing the validity of labeled emotion revealed that labeled emotion was significantly distinguishable from non-labeled emotion in the “anger,” “happiness,” “peacefulness,” “sadness,” and “solemnness” categories (Table 4). However, the “gentleness” emotion was indistinguishable from the “happiness” and “peacefulness” emotions in the “gentleness” category, and the “transcendence” emotion was easily confused with the “peacefulness” emotion in the “transcendence” category. This may be due to the high correlation between these variables.

The relationship between the dimensional and discrete emotion models was explored. In order to generate results comparable to those in previous studies, we temporarily binned valence and arousal into nine categories (Valence: positive, neutral, negative × Arousal: low, moderate, high). The arousal and valence ratings fit well with the discrete emotion representations. As seen in Table 5, “happiness” conveys positive and highly arousing emotion, and “sadness” conveys negative and moderately arousing emotion, consistent with the results in many previous studies, regardless of the stimuli used (e.g., word, short text, or face) (Bradley & Lang, 1999; Eerola & Vuoskoski, 2011; Darcy & Fontaine, 2020; Hinojosa et al., 2016; Langner et al., 2010; Paquette et al., 2013; Stevenson et al., 2007; Vieillard et al., 2008). Moreover, “happiness” is the only discrete emotion classified as positive, consistent with the IADS (International Affective Digitized Sounds), in which “happiness” is only significantly predictive for positive sounds (Stevenson & James, 2008).

Previous studies using word (Schimmack & Grob, 2000; Syssau et al., 2021), text (Imbir, 2016), face (Prada et al., 2018), voice (Paquette et al., 2013; Juslin & Laukka, 2003), and Western music (Eerola & Vuoskoski, 2011; Fuentes‐Sánchez et al., 2021; Shen et al., 2018) as stimuli reported “anger” as a negative and highly arousing emotion. However, the present study shows that the “anger” excerpt conveyed neutral and highly arousing emotion. Wang et al. (2021) reported a consistent result in which “anger” in Chinese traditional music is a neutral and highly arousing emotion. Such a result indicates that Chinese traditional music has distinct emotional characteristics in the angry dimension. In particular, Chinese traditional music mainly uses a fast rhythm feature to convey anger (Wang et al., 2022), while Western music utilizes unstable harmony to express the feeling of nervousness (Bai et al., 2016).

In previous studies using Western music, neutral, tenderness, and peacefulness music were found to represent neither positive nor negative valence type (Paquette et al., 2013; Prada et al., 2018; Eerola & Vuoskoski, 2011; Fuentes-Sánchez et al., 2021; Wang et al., 2021). Consistently, in the present study, while the “gentleness” and “solemnness” excerpts were moderately arousing and the “peacefulness” and “transcendence” ones less arousing, they all demonstrated neutral valence. The result that “gentleness” expressed neutral emotion contradicts our expectation, as “gentleness” should be a positive valence type. This may be due to the small sample size (*n* = 3) of typical gentle excerpts in our dataset.

The intensity rating of “happiness” was the highest among the seven discrete emotions (Table 5). This might be because of the highly arousing attribute of the “happiness” emotion. Prada et al. (2018) and Eerola and Vuoskoski (2011) similarly reported that high-arousal emotions such as happiness, anger, and fear have higher intensity ratings. Furthermore, our result showed that familiarity significantly enhanced the valence, arousal, and intensity ratings for the “happiness” emotion, consistent with previous studies showing that familiarity enhances music-evoked pleasure (Morris et al., 2019). It has been reported that “familiarity” not only plays a role in emotional responses to music, but also improves emotion recognition (Filipic et al., 2010; Freitas et al., 2018; Laukka et al., 2013; Susino & Schubert, 2018). It remains to be addressed whether and how familiarity affects emotion recognition of Chinese traditional music remain.

The music excerpts in the present study were classified into four subsets, using cluster analysis (Fig. 6). We found that the optimal number of parameters obtained by the k-means clusters was four rather than seven. This might be because the discrete emotion model we used had a strong correlation between some variables. Upon analyzing the score values of the discrete emotions in each cluster, we found that the emotion labels of the first two highest ratings played a decisive role in k-means classification, suggesting that music emotions are complex and multidimensional, and a piece of music could convey multiple emotions, making it difficult to define music with only one emotion label. Indeed, previous studies found that listeners may experience both sadness and happiness at the same time when exposed to stimuli with mixed emotional cues (Hunter et al., 2008; Larsen & McGraw, 2011). In the IADS, researchers labeled auditory stimuli with single or dual discrete emotion labels based on emotion rating with one standard deviation higher (Stevenson & James, 2008). In the emotional film music dataset, researchers obtained highly and moderately typical music examples based on the rank of typicality, where the typicality value was calculated using the following formula: [the mean of the rating on target emotion − the mean of the ratings on non-target emotions − the standard deviation of the target emotion rating] (Eerola & Vuoskoski, 2011).

The present study found that the familiarity variable was the only factor affected by the participant's sex in rating. In previous studies using Western music, conclusions about sex differences are somewhat controversial. For example, Guedes et al. (2023) found that male participants provided higher “surprise” ratings than female participants; Paquette et al. (2013) reported that male participants gave slightly higher “intensity” ratings; Li et al. (2022) reported no sex difference in rating valence, arousal, and dominance; and Stevenson et al. (2007) found that female participants gave higher ratings than male participants for all negative emotions. The discrepancy regarding sex differences remains to be addressed.

Our dataset possesses some obvious advantages. Firstly, instead of only one emotion model, both dimensional and discrete emotion models were used to assess the emotions of the music excerpts. Thus, our dataset offers both valence and arousal scores, making it comparable to previous studies, while providing domain-specific discrete emotion ratings to describe the unique affective emotions of Chinese traditional music. Secondly, multiple discrete emotion ratings for each excerpt, instead of only one emotion category, were evaluated, and k-means clustering was employed to classify the discrete emotions. Hence, the multidimensional and complex nature of music emotions could be revealed. Thirdly, a familiarity variable was also included, and a significant emotion-enhancing effect was found in the “happiness” category, suggesting that familiarity should be controlled in studying music emotions. Finally, a sufficient sample of participants was recruited to evaluate the music excerpts, and good reliability was found among the rating variables.

However, the study has a few shortcomings. Firstly, although our dataset contains more music excerpts than most other music sets (Belfi & Kacirek, 2021; Eerola & Vuoskoski, 2011; Imbir, 2016; Paquette et al., 2013; Stevenson & James, 2008; Vieillard et al., 2008), the number of excerpts is not balanced among various conditions. The number of excerpts is relatively small in some subcategories such as familiar, anger, gentleness, solemnness, and transcendence. Secondly, the low-level auditory properties of the excerpts were not controlled. The music materials we selected come from naturalistic music with high ecological validity, but their acoustic characteristics are relatively diverse and we could only control limited dimensions such as loudness, duration, and amplitude. Thirdly, the assessments were carried out on young Chinese college students. The effectiveness of the musical excerpts should be further evaluated in participants of different ages and cultural and social backgrounds.

Future work should be dedicated to (1) enriching the materials of the dataset to ensure that each category has a relatively large number of excerpts; (2) investigating the low-level auditory properties of the excerpts and their connection to emotions, as previous studies have shown that timbre (Eerola & Vuoskoski, 2012; Kraus et al., 2009; Panda et al., 2020), rhythm (Bispham, 2006; Trost et al., 2017; Yan et al., 2019), and pitch (Frick, 1985; Jaquet et al., 2014; Schellenberg et al., 2000) are strongly related to music emotions; (3) re-assessing the validity and reliability of the dataset across cultural contexts and social identities; and (4) investigating the brain mechanisms underlying the perception, memory, and emotional responses for Chinese traditional instrumental music using the dataset.

## Summary and conclusion

The present study constructed a Chinese traditional instrumental music dataset and provided rating data for each music excerpt on ten variables: familiarity, dimensional emotions (valence and arousal) and discrete emotions (anger, gentleness, happiness, peacefulness, sadness, solemnness, and transcendence). We believe that the dataset is a useful tool and could contribute to cross-cultural studies on emotional responses of music.

### Supplementary Information

Below is the link to the electronic supplementary material.

Supplementary file1 (DOC 468 KB)

## Declarations

### Open practices statement

All data and stimuli, including supplementary figures and tables, can be found at: https://osf.io/tzkx7/?view_only=c31867bc2ddf413583286bc0a582635a. The present work was not preregistered.
