# Supplementary material for: Construction of a Chinese traditional instrumental music dataset: A validated set of naturalistic affective music excerpts
Source: Behav Res Methods. 2024 May 3;56(4):3757–78. doi: 10.3758/s13428-024-02411-6 (PMC11133124; doi:10.3758/s13428-024-02411-6)
Supplement: Supplementary file 1 — Supplementary file1 (DOC 468 KB) [file 13428_2024_2411_MOESM1_ESM.doc]

**Supplementary Information for**

**Construction of Chinese Traditional Instrumental Music Dataset: A Validated Set of Naturalistic Affective Music Excerpts**

Di Wu^1,2^, Xi Jia^1,2^, Wenxin Rao^1^, Wenjie Dou^1,2^, Yangping Li^1,3^, Baoming Li^1,2^

*^1^ Institute of Brain Science and Department of Physiology, School of Basic Medical Sciences, Hangzhou Normal University, Hangzhou, 311121, China*

*^2^ Zhejiang Philosophy and Social Science Laboratory for Research in Early Development and Childcare, Hangzhou Normal University, Hangzhou, 311121, China*

*^3^ School of Foreign Studies, Xi’an Jiaotong University, Xi’an, 710049, China*

**Correspondence Author**

Baoming Li, PhD and Professor

Institute of Brain Science and Department of Physiology,

School of Basic Medical Sciences,

Hangzhou Normal University

Email: bmli@hznu.edu.cn

**This file includes:**

1. Correlation Analysis: Familiarity with Valence, Arousal, and Labeled-emotion Intensity of each Music Emotion Category

2. Correlation Analysis: Between Dimensional and Discrete Emotional Models

3. Result for Experiment 2

4. Educational Qualification of the Participants

**1. Correlation Analysis: Familiarity with Valence, Arousal, and Emotion Intensity of each Music Emotion Category**

We have conducted the person correlation analysis in which familiarity using familiarity as a continuous variable to assess its impact on emotional perception. We have conducted Pearson correlation analysis, in which 'familiarity' was used as a continuous variable, and revealed a significant moderate to strong positive correlations between familiarity and valence, arousal or intensity of labeled emotion within the happiness music category (see Supplementary Table 1). This result is consistent with the conclusion based on the familiar vursus unfamiliar comparison, indicating that familiarity enhances emotional perception in happy music.

In addition, Pearson correlation analysis revealed a significant moderate negative correlation between familiarity and arousal in the transcendence music category (see Supplementary Table 1), suggesting that familiarity potentially enhances emotional experiences associated with music aesthetics.

***Supplementary Table 1***. Correlation of familiarity with valence, arousal and labeled-emotion intensity

| **Category** | **Correlation** | | |
| --- | --- | --- | --- |
|  | **Familiarity**  **vs.**  **Valence** | **Familiarity**  **vs.**  **Arousal** | **Familiarity**  **vs.**  **Labeled-emotion Intensity** |
| Anger | 0.26 | 0.34 | 0.17 |
| Gentleness | -0.93 | 0.62 | -0.46 |
| Happiness | 0.67^***^ | 0.56^***^ | 0.63^***^ |
| Peacefulness | 0.29 | 0.10 | 0.16 |
| Sadness | 0.28 | 0.07 | -0.07 |
| Solemnness | 0.30 | 0.03 | -0.16 |
| Transcendence | -0.52 | -0.58^*^ | 0.50 |

*p<.05; **p<.01; ***p<.001.

**2. Correlation Analysis: Between Dimensional and Discrete Emotional Models**

We have conducted correlations between dimensions and discrete emotions as presented in Supplementary Table 2. 'happiness' and 'gentleness' both displayed a strong positive correlation with valence, while 'sadness' exhibited a strong negative correlation with valence. The remaining discrete emotions (except 'anger'), which was classified as neutral, displayed a weak to moderate negative correlation with valence. The correlation analysis also revealed that 'angry', 'gentleness' and 'happiness' all had a moderate to strong positive correlations with arousal, while the 'peacefulness', 'sadness', 'solemnness' and 'transcendence' a moderate to strong negative correlation with arousal.

These correlation results were consistent with the results revealed by analyzing the positions of diverse discrete emotional music categories within the valence-arousal quadrant (see Figure 3). For instance, emotions categorized as positive valence and high arousal, such as 'happiness', exhibited a strong positive correlation with both valence and arousal. Moreover, emotions classified as having neutral valence or moderately arousal demonstrated varying degrees of correlation with valence and arousal, suggesting that some neutral emotions for music is still associated with emotional experience.

***Supplementary Table 2. Correlation Matrix Between Dimensional and Discrete emotions***

| **Correlation** | ***Discrete Model*** | | | | | | |
| --- | --- | --- | --- | --- | --- | --- | --- |
|  | Anger | Gentleness | Happiness | Peacefulness | Sadness | Solemnness | Transcendence |
| ***Dimensional Model*** |  |  |  |  |  |  |  |
| Valence | 0.02 | 0.81^***^ | 0.95^***^ | -0.21^***^ | -0.91^***^ | -0.53^***^ | -0.44^***^ |
| Arousal | 0.48^***^ | 0.52^***^ | 0.84^***^ | -0.72^***^ | -0.61^***^ | -0.43^***^ | -0.80^***^ |

*p<.05; **p<.01; ***p<.001.

**3. Result for Experiment 2**

We have performed a new experiment (Experiment 2) to verify the validation of the discrete emotional label derived from the dimension with the highest mean rating, and the result is presented in *Supplementary Table 3*. In this study, a total of 30 students (15 females; 15 males) from Hangzhou Normal University were recruited (M_years_=20.64, SD_years_=1.93, Range=[19, 27]). The music stimuli were divided into 3 lists, each evaluated by 10 participants.

The experimental procedure involved participants listening to music and engaging in an emotional force-choice task and seven emotional rating tasks. For each trial, an excerpt was played once on the first screen and participants were required to complete a force-choice task in which chose the most suitable emotion they think this music represents among seven discrete emotions (anger, gentleness, happiness, peacefulness, sadness, solemnness and transcendence). Then, the second screen where the excerpts were played once again, and participants were required to rate seven categories of discrete emotion for the excerpt using the seven-point Likert scale.

We compared consistency between the emotion chosen in the forced-choice task and the maximum emotional rating across the seven emotional dimensions for each participant. The resulting consistency rate was 97.03%. This indicates that, for individual participants, the maximum intensity score aligns with the single best-fitting category label.

The overall accuracy, indicating correspondence between the forced-choice emotions in Experiment 2 and the maximal mean ratings in Experiment 1, is 52.86%. The recognition rates of emotions such as anger, happiness, and sadness are relatively higher, around 60%, whereas neutral emotions like peacefulness, transcendence, solemnness, and gentleness demonstrate lower recognition rates, only around 30%. This might be attributed to the interrelated nature of these neutral emotions, leading participants to easily confuse them when making emotional force choice. The recognition rates in our study are lower compared to some Western music studies (Argstatter, 2015; Castro et al., 2011; Paquette et al., 2013; Lévêque et al., 2018), possibly due to inherent characteristics of Chinese traditional music.

The percentages of emotional classification for each discrete emotional music category were computed. As shown in Supplementary Table 3, the intended emotion received the highest recognition rate compared to other emotions, except for the gentleness category (which contained a small sample size). This result validates that the emotional label obtained through the maximal mean rating dimension indeed represents the most suitable emotion expressed by a specific music excerpt.

We reprocessed the data of Experiment 1 by identifying the 'force choice emotion(s)' in Experiment 1 as the maximal rating of emotional dimension(s) assessed by each participant, following a methodology mentioned by Vieillard et al. (2008). We also analyzed the percentages of emotional classification for each distinct emotional music category, and the result is in line with that in Experiment 2 (see *Supplementary Table 3*).

***Supplementary Table 3***. The percentage of emotional classification for each music category in Experiments 2 and 1

| **Category**  **Classification** | **Anger** | **Gentleness** | **Happiness** | **Peacefulness** | **Sadness** | **Solemnness** | **Transcendence** |
| --- | --- | --- | --- | --- | --- | --- | --- |
| ***Experiment 2*** |  |  |  |  |  |  |  |
| Anger | 62.5 | 1.7 | 10.8 | 1.7 | 8.3 | 12.5 | 2.5 |
| Gentleness | 0.0 | 26.7 | 26.7 | 33.3 | 6.7 | 0.0 | 6.7 |
| Happiness | 9.8 | 11.6 | 63.9 | 8.0 | 3.2 | 1.7 | 1.9 |
| Peacefulness | 0.8 | 12.9 | 4.8 | 38.8 | 17.1 | 7.1 | 18.5 |
| Sadness | 2.0 | 4.1 | 4.4 | 13.1 | 58.6 | 9.4 | 8.5 |
| Solemnness | 7.1 | 3.8 | 3.8 | 11.9 | 24.3 | 31.4 | 17.6 |
| Transcendence | 2.6 | 1.6 | 3.2 | 28.9 | 16.3 | 11.1 | 36.3 |
|  |  |  |  |  |  |  |  |
| ***Experiment 1*** |  |  |  |  |  |  |  |
| Anger | 60.1 | 2.1 | 19.0 | 3.7 | 8.5 | 21.6 | 6.8 |
| Gentleness | 0.0 | 34.5 | 33.3 | 32.1 | 13.7 | 4.2 | 11.3 |
| Happiness | 7.7 | 19.7 | 73.8 | 9.4 | 5.1 | 4.3 | 6.2 |
| Peacefulness | 1.1 | 15.5 | 11.1 | 46.2 | 21.6 | 11.4 | 28.1 |
| Sadness | 5.0 | 6.8 | 5.4 | 17.5 | 61.5 | 14.1 | 14.2 |
| Solemnness | 6.5 | 4.0 | 5.4 | 17.5 | 27.9 | 47.1 | 20.7 |
| Transcendence | 2.8 | 3.7 | 3.8 | 32.4 | 25.2 | 23.0 | 45.6 |

**4. Educational Qualification of the Participants**

| Participant Number | Education  Level | Major | Sex | Age | Music List |
| --- | --- | --- | --- | --- | --- |
| ***Experiment 1*** |  |  |  |  |  |
| 1 | Undergraduate | Economics | Female | 21 | 2 |
| 2 | Undergraduate | Computer Science | Female | 21 | 2 |
| 3 | Undergraduate | Food Quality and Safety | Female | 21 | 2 |
| 4 | Postgraduate | Psychology | Female | 23 | 2 |
| 5 | Undergraduate | Economics | Female | 22 | 2 |
| 6 | Undergraduate | Scientific Education | Female | 23 | 2 |
| 7 | Undergraduate | Psychology | Female | 24 | 2 |
| 8 | Postgraduate | Clinical Medicine | Female | 20 | 2 |
| 9 | Undergraduate | Psychology | Female | 24 | 2 |
| 10 | Postgraduate | Mathematics | Female | 21 | 2 |
| 11 | Undergraduate | Mathematics | Female | 22 | 2 |
| 12 | Undergraduate | Mathematics | Female | 22 | 2 |
| 13 | Undergraduate | Chemistry | Male | 23 | 3 |
| 14 | Undergraduate | Chemistry | Male | 21 | 3 |
| 15 | Undergraduate | Chemistry | Female | 22 | 3 |
| 16 | Undergraduate | Movie | Female | 22 | 3 |
| 17 | Postgraduate | Chemistry | Female | 21 | 3 |
| 18 | Undergraduate | Chinese Language and Literature | Female | 21 | 3 |
| 19 | Undergraduate | Chinese Language and Literature | Female | 22 | 3 |
| 20 | Undergraduate | Internet of things | Female | 22 | 3 |
| 21 | Undergraduate | Chinese Language and Literature | Female | 22 | 3 |
| 22 | Undergraduate | Chinese Language and Literature | Female | 21 | 3 |
| 23 | Undergraduate | Chinese Language and Literature | Female | 21 | 3 |
| 24 | Undergraduate | Chinese Language and Literature | Female | 21 | 3 |
| 25 | Undergraduate | Mathematics | Female | 18 | 3 |
| 26 | Undergraduate | Preventive Medicine | Female | 22 | 3 |
| 27 | Undergraduate | Preschool Education | Female | 22 | 3 |
| 28 | Undergraduate | Preschool Education | Female | 22 | 3 |
| 29 | Undergraduate | Chemistry | Female | 22 | 3 |
| 30 | Undergraduate | Chemistry | Female | 24 | 3 |
| 31 | Postgraduate | Psychology | Male | 24 | 3 |
| 32 | Postgraduate | Chinese Language and Literature | Female | 24 | 3 |
| 33 | Postgraduate | International Chinese Education | Female | 24 | 1 |
| 34 | Undergraduate | Physic Education | Male | 20 | 1 |
| 35 | Undergraduate | Computer Science | Male | 24 | 1 |
| 36 | Undergraduate | Computer Science | Female | 22 | 1 |
| 37 | Undergraduate | Physical Education | Male | 23 | 1 |
| 38 | Postgraduate | Jurisprudence | Female | 24 | 1 |
| 39 | Undergraduate | Primary Education | Female | 20 | 1 |
| 40 | Postgraduate | Primary Education | Female | 23 | 1 |
| 41 | Undergraduate | Clinical Medicine | Female | 20 | 1 |
| 42 | Undergraduate | Logistics Management | Female | 20 | 1 |
| 43 | Postgraduate | Health Policy and Management | Female | 23 | 1 |
| 44 | Undergraduate | Computer Science | Female | 23 | 1 |
| 45 | Undergraduate | Mathematics | Male | 24 | 1 |
| 46 | Postgraduate | Physical Education | Male | 25 | 1 |
| 47 | Undergraduate | Electronic Commerce | Male | 21 | 2 |
| 48 | Postgraduate | Economics | Male | 26 | 2 |
| 49 | Undergraduate | Biology | Male | 22 | 3 |
| 50 | Postgraduate | Chemistry | Female | 25 | 3 |
| 51 | Undergraduate | Chinese Language | Male | 21 | 3 |
| 52 | Postgraduate | Economics | Female | 26 | 3 |
| 53 | Postgraduate | Health Management | Female | 25 | 1 |
| 54 | Undergraduate | Computer Science | Female | 22 | 1 |
| 55 | Undergraduate | Economics | Female | 22 | 1 |
| 56 | Postgraduate | Economics | Female | 24 | 1 |
| 57 | Undergraduate | Jurisprudence | Female | 26 | 1 |
| 58 | Postgraduate | Jurisprudence | Female | 24 | 1 |
| 59 | Postgraduate | Jurisprudence | Female | 23 | 1 |
| 60 | Undergraduate | Mega Data | Female | 20 | 1 |
| 61 | Postgraduate | Jurisprudence | Male | 23 | 2 |
| 62 | Undergraduate | Preschool Education | Female | 22 | 2 |
| 63 | Undergraduate | Health policy and Management | Male | 22 | 2 |
| 64 | Undergraduate | English | Male | 21 | 2 |
| 65 | Postgraduate | Primary Education | Female | 23 | 1 |
| 66 | Postgraduate | Primary Education | Female | 24 | 1 |
| 67 | Postgraduate | Contemporary Chinese Literature | Female | 24 | 1 |
| 68 | Undergraduate | Preventive Medicine | Female | 21 | 1 |
| 69 | Postgraduate | Health Policy and Management | Female | 23 | 2 |
| 70 | Undergraduate | Preschool Education | Female | 22 | 2 |
| 71 | Undergraduate | Digital Media Art | Female | 22 | 2 |
| 72 | Postgraduate | Administration | Female | 23 | 2 |
| 73 | Postgraduate | Classical Chinese Literature | Female | 28 | 2 |
| 74 | Undergraduate | Preventive Medicine | Female | 18 | 2 |
| 75 | Undergraduate | English | Female | 23 | 2 |
| 76 | Postgraduate | Chemistry | Male | 25 | 2 |
| 77 | Undergraduate | Scientific Education | Male | 22 | 2 |
| 78 | Postgraduate | Chinese History | Female | 23 | 2 |
| 79 | Postgraduate | Chinese History | Female | 22 | 2 |
| 80 | Postgraduate | Chinese History | Female | 24 | 2 |
| 81 | Postgraduate | Chinese Education | Female | 24 | 2 |
| 82 | Postgraduate | Electronic Commerce | Female | 24 | 2 |
| 83 | Postgraduate | Chinese History | Male | 26 | 2 |
| 84 | Postgraduate | Nursing | Female | 24 | 2 |
| 85 | Undergraduate | Internet of Things | Female | 20 | 1 |
| 86 | Undergraduate | Nursing | Female | 20 | 1 |
| 87 | Postgraduate | English | Male | 24 | 1 |
| 88 | Postgraduate | Contemporary Chinese Literature | Male | 24 | 1 |
| 89 | Undergraduate | Nursing | Female | 25 | 1 |
| 90 | Undergraduate | Preschool Education | Female | 21 | 1 |
| 91 | Undergraduate | Biotechnology | Female | 21 | 1 |
| 92 | Undergraduate | Biotechnology | Female | 21 | 1 |
| 93 | Undergraduate | Biotechnology | Female | 22 | 1 |
| 94 | Postgraduate | Japanese | Male | 23 | 1 |
| 95 | Undergraduate | Psychology | Female | 20 | 3 |
| 96 | Undergraduate | Biotechnology | Female | 21 | 3 |
| 97 | Postgraduate | Chinese Modern Economics History | Male | 23 | 1 |
| 98 | Undergraduate | Nursing | Female | 21 | 3 |
| 99 | Undergraduate | Clinical Medicine | Female | 20 | 3 |
| 100 | Postgraduate | Bibliography | Male | 33 | 1 |
| 101 | Undergraduate | Primary Education | Female | 21 | 3 |
| 102 | Postgraduate | Classical Chinese Literature | Male | 23 | 1 |
| 103 | Postgraduate | Psychology | Male | 25 | 3 |
| 104 | Postgraduate | Chemical Industry | Male | 24 | 3 |
| 105 | Undergraduate | Mathematics | Female | 21 | 2 |
| 106 | Undergraduate | Nursing | Female | 21 | 2 |
| 107 | Postgraduate | Developmental Biology | Male | 24 | 3 |
| 108 | Undergraduate | English | Female | 21 | 3 |
| 109 | Undergraduate | Preventive Medicine | Female | 19 | 3 |
| 110 | Undergraduate | Clinical Medicine | Male | 21 | 3 |
| 111 | Undergraduate | Jurisprudence | Male | 22 | 3 |
| 112 | Undergraduate | Nursing | Male | 21 | 3 |
| 113 | Undergraduate | Computer Science | Female | 20 | 3 |
| 114 | Undergraduate | Computer Science | Female | 21 | 3 |
| 115 | Undergraduate | Nursing | Male | 22 | 3 |
| 116 | Undergraduate | Jurisprudence | Male | 22 | 3 |
| 117 | Postgraduate | Electronic and Information Engineering | Male | 26 | 2 |
| 118 | Postgraduate | Electronic and Information Engineering | Male | 25 | 1 |
| 119 | Undergraduate | Biology | Male | 22 | 2 |
| 120 | Undergraduate | Biology | Male | 21 | 2 |
| 121 | Undergraduate | Chemical Industry | Male | 21 | 2 |
| 122 | Undergraduate | Chemical Industry | Male | 21 | 2 |
| 123 | Postgraduate | Psychology | Male | 24 | 2 |
| 124 | Postgraduate | Psychology | Male | 24 | 2 |
| 125 | Postgraduate | Psychology | Male | 28 | 2 |
| 126 | Undergraduate | Health Management | Male | 20 | 2 |
| 127 | Postgraduate | Chemical Industry | Male | 24 | 2 |
| 128 | Postgraduate | Psychology | Male | 24 | 2 |
| 129 | Undergraduate | Primary Education | Male | 20 | 2 |
| 130 | Undergraduate | Primary Education | Male | 20 | 2 |
| 131 | Postgraduate | Psychology | Male | 24 | 2 |
| 132 | Undergraduate | Digital Media Art | Male | 21 | 3 |
| 133 | Postgraduate | Psychology | Male | 23 | 3 |
| 134 | Undergraduate | Preschool Education | Male | 21 | 3 |
| 135 | Postgraduate | Psychology | Male | 25 | 1 |
| 136 | Undergraduate | Computer Science | Male | 24 | 1 |
| 137 | Undergraduate | Computer Science | Male | 21 | 1 |
| 138 | Postgraduate | Psychology | Male | 26 | 1 |
| 139 | Postgraduate | Chemical Industry | Male | 23 | 2 |
| 140 | Postgraduate | Movie | Male | 26 | 1 |
| 141 | Postgraduate | Psychology | Male | 22 | 1 |
| 142 | Postgraduate | Psychology | Male | 24 | 1 |
| 143 | Undergraduate | Preventive Medicine | Male | 18 | 1 |
| 144 | Undergraduate | Computer Science | Male | 20 | 2 |
| 145 | Undergraduate | Clinical Medicine | Male | 20 | 2 |
| 146 | Undergraduate | Computer Science | Male | 21 | 2 |
| 147 | Postgraduate | Sociology | Male | 25 | 1 |
| 148 | Undergraduate | Computer Science | Male | 21 | 2 |
| 149 | Postgraduate | Psychology | Male | 26 | 3 |
| 150 | Postgraduate | Psychology | Male | 26 | 3 |
| 151 | Undergraduate | Psychology | Male | 20 | 3 |
| 152 | Postgraduate | Chemical Industry | Male | 24 | 1 |
| 153 | Undergraduate | Pharmacy | Male | 20 | 1 |
| 154 | Undergraduate | Computer Science | Male | 19 | 3 |
| 155 | Undergraduate | Biology | Male | 20 | 3 |
| 156 | Undergraduate | Economics | Male | 24 | 3 |
| 157 | Undergraduate | Chinese Language | Male | 22 | 3 |
| 158 | Undergraduate | Preventive Medicine | Male | 19 | 3 |
| 159 | Undergraduate | Preventive Medicine | Male | 18 | 3 |
| 160 | Undergraduate | Computer Science | Male | 19 | 3 |
| 161 | Undergraduate | Biology | Male | 19 | 3 |
| 162 | Undergraduate | Public art | Male | 23 | 1 |
| 163 | Undergraduate | Chemistry | Male | 21 | 1 |
| 164 | Postgraduate | Psychology | Male | 24 | 1 |
| 165 | Undergraduate | Computer Science | Male | 21 | 2 |
| 166 | Undergraduate | Nursing | Male | 20 | 2 |
| 167 | Undergraduate | Health Policy and Management | Male | 22 | 1 |
| 168 | Undergraduate | Health Policy and Management | Male | 21 | 1 |
|  |  |  |  |  |  |
| ***Experiment 2*** |  |  |  |  |  |
| 1 | Undergraduate | Electronic and Information Engineering | Male | 21 | 1 |
| 2 | Undergraduate | Biotechnology | Male | 20 | 2 |
| 3 | Postgraduate | Physiology | Male | 27 | 1 |
| 4 | Undergraduate | Clinical Medicine | Female | 19 | 3 |
| 5 | Undergraduate | Clinical Medicine | Female | 20 | 1 |
| 6 | Undergraduate | Clinical Medicine | Female | 20 | 3 |
| 7 | Undergraduate | Clinical Medicine | Female | 20 | 2 |
| 8 | Undergraduate | Animation | Male | 22 | 1 |
| 9 | Undergraduate | Clinical Medicine | Male | 19 | 1 |
| 10 | Undergraduate | Clinical Medicine | Male | 19 | 2 |
| 11 | Undergraduate | Mathematics | Male | 20 | 3 |
| 12 | Undergraduate | Clinical Medicine | Female | 19 | 1 |
| 13 | Undergraduate | Physics | Female | 20 | 2 |
| 14 | Postgraduate | Physiology | Male | 25 | 3 |
| 15 | Undergraduate | Pharmacy | Female | 20 | 1 |
| 16 | Undergraduate | Pharmacy | Female | 20 | 1 |
| 17 | Undergraduate | Pharmacy | Male | 19 | 2 |
| 18 | Undergraduate | Nursing | Female | 20 | 3 |
| 19 | Postgraduate | Primary Education | Female | 22 | 1 |
| 20 | Postgraduate | Primary Education | Female | 23 | 2 |
| 21 | Undergraduate | Economics | Male | 21 | 1 |
| 22 | Postgraduate | Chemistry | Male | 24 | 3 |
| 23 | Undergraduate | Chemistry | Female | 19 | 2 |
| 24 | Undergraduate | Chemistry | Female | 19 | 2 |
| 25 | Undergraduate | Primary Education | Female | 20 | 3 |
| 26 | Undergraduate | Chemistry | Female | 19 | 3 |
| 27 | Undergraduate | Nursing | Male | 19 | 2 |
| 28 | Undergraduate | Preventive Medicine | Male | 20 | 3 |
| 29 | Undergraduate | Geographical Information Science | Male | 21 | 2 |
| 30 | Postgraduate | Physiology | Male | 22 | 3 |
